# Chronic exposure to a neonicotinoid increases expression of antimicrobial peptide genes in the bumblebee *Bombus impatiens*

**DOI:** 10.1038/srep44773

**Published:** 2017-03-21

**Authors:** William R. Simmons, David R. Angelini

**Affiliations:** 1 Department of Biology, Colby College, 5734 Mayflower Hill, Waterville, ME, 04901, USA; 2 National Human Genome Research Institute, 49 Convent Drive, Bethesda, MD, 20892, USA

## Abstract

Bumblebees are important pollinators in wild and agricultural settings. In recent decades pollinator declines have been linked to the effects of increased pesticide use and the spread of disease. Synergy between these factors has been suggested, but no physiological mechanism has been identified. This study examines the connection between neonicotinoid exposure and innate immune function in the bumblebee *Bombus impatiens*, which is an important wild and commercial pollinator in eastern North America. Experimental colonies in the field were enclosed and provided pollen and sugar syrup containing an agriculturally relevant range of imidacloprid concentrations. Bumblebees were collected from colonies over four weeks, and the expression of antimicrobial peptides was measured using multiplex quantitative real time PCR. Significant increases in the expression of *abaecin, apidaecin* and *hymenoptaecin* were found over time in treatments receiving moderate to high concentrations of the pesticide. Responses were dependent on time of exposure and dose. These results indicate that immune function in bumblebees is affected by neonicotinoid exposure and suggest a physiological mechanism by which neonicotinoids may impact the innate immune function of bumblebee pollinators in wild and agricultural habitats.

Pollination is a critical ecosystem service, essential for the persistence of wild habitats as well as human agriculture^1^. The rapid growth in agricultural demand means that managed pollinator species, such as honeybees, are unlikely to meet the demand for agricultural pollination on their own^2^, increasing the importance of pollination by wild insects^3^. Bumblebees play a key role in pollination, but are experiencing widespread declines in wild populations^4^,^5^. The cause of these declines has been the focus of much attention, and may in fact be multi-factorial^6^,^7^. Some of the potential influences that have been suggested include the spread of parasites as well as exposure to pesticides. In particular, neonicotinoid pesticides have been a focus of concern^8^. Because of the critical role of wild insect pollinators and the danger posed by their decline, greater understanding of their biological response to these factors is imperative.

Neonicotinoids have been shown to negatively affect bees in several ways, and collectively it is thought that these off-target effects limit the effectiveness of commercial and natural pollination services. Since they are systemic, neonicotinoids or their metabolites can be present in the nectar and pollen of plants after application, with the potential for chronic exposure to pollinators^9^,^10^,^11^,^12^,^13^,^14^. Neonicotinoids are neurotoxic and have been found to alter foraging behavior, reduce foraging efficiency^15^,^16^,^17^,^18^, and increase displacement of queens in honeybees^19^. Similar effects of neonicotinoid exposure have been reported in the European buff-tailed bumblebee *Bombus terrestris*^20^,^21^. Exposure has also been linked to reduced colony growth and reproduction of *B. terrestris*^9^,^22^,^23^,^24^. Individual survival of *B. terrestris* is also lower as a result of neonicotinoid treatment^9^,^23^,^25^,^26^. A recent study of the North American common eastern bumblebee *Bombus impatiens* found similar negative effects for two neonicotinoids on queen mortality and foraging efficiency^27^. Furthermore, pathogen challenge experiments using honeybees exposed to neonicotinoids resulted in increased levels of *Nosema*^28^ and deformed wing virus^29^. However, at low concentrations (1 ppb clothianidin), effects on learning, fecundity and disease susceptibility may vary^30^.

Despite previous work characterizing neonicotinoid effects on health, there has been relatively little investigation of mechanisms by which neonicotinoids might increase bees’ susceptibility to infection^31^. One possibility is that increased rates of infection following neonicotinoid exposure might be explained by suppression of individual insects’ innate immune system^21^ or of a colony’s social immunity. Several recent studies have addressed this possibility in honeybees. Alaux and colleagues^6^ found that honeybee mortality resulting from *Nosema* infection increased significantly with exposure to imidacloprid, a neonicotinoid commonly used in agriculture. In their study, phenoloxidase activity and hemocyte numbers, reflecting individual immunity, were not altered. However glucose oxidase, which functions in colony-level immunity, was significantly reduced in treatments receiving both *Nosema* and imidacloprid, suggesting a synergistic effect. Brandt and coworkers^32^ found that exposure to a similar concentration of imidacloprid, as well as to the neonicotinoids thiacloprid and clothianidin, did suppress individual immunity, as measured by hemocyte numbers, foreign-body encapsulation, and the antimicrobial activity of hemolymph after 24 hours. The authors suggest the differences in their results may stem from differing responses of healthy bees versus those with prior exposure to *Nosema*.

Antimicrobial peptides (AMPs) are a major component in insect innate immunity and exist in a diverse range of organisms^33^. The AMPs of insects have been considered most effective against Gram-positive bacteria, with more limited activity against Gram-negative bacteria and fungi^34^,^35^. Wounding and infection with *E. coli* cause increased expression of AMPs in *B. terrestris*^36^. Another recent study of this bumblebee species demonstrated that the AMPs *abaecin* and *defensin* were also up-regulated during infection with the trypanosome *Crithidia bombi*. Suppression of these genes with RNA interference also led to increased parasite titers^37^. Similarly, exposure of honeybees to Varroa mites increased AMP expression^38^, although this may have been due to secondary viral infection. AMP expression in honeybees is down-regulated after infection with *Nosema ceranae*, however this parasite is thought to suppress host immunity^39^.

Work with honeybee larvae has begun to explore potential influences of neonicotinoid exposure on AMP expression. Gregorc and colleagues^38^ examined the effects of a high imidacloprid concentration on the expression of 23 immunity-related genes, finding increased expression of prophenoloxidase-activating enzyme, but no significant effects on transcript levels for five AMP genes. That study also found no synergistic effects of pesticide exposure and mite parasitism of larvae. Di Prisco and coworkers^29^ examined the effects of multiple neonicotinoids on honeybees and reported that clothianidin treatment caused up-regulation of an inhibitor of NF-κB, as well as reduced expression of the AMP *apidaecin*. In both these studies, organophosphate insecticides did not produce comparable changes in gene expression. Aufauvre and coworkers^40^ examined the influences of imidacloprid exposure and *Nosema ceranae* infection on honeybee workers, finding reduced expression of *hymenoptaecin*, alone among AMPs, after exposure to both the pesticide and parasite.

No study to date has tested whether neonicotinoids can alter the expression of antimicrobial peptide genes in bumblebees. Therefore, the goal of this study was to test this hypothesis by examining the expression of four AMPs in the common eastern bumblebee *Bombus impatiens*. This was done over one month, following exposure to a range of agriculturally realistic concentrations of the neonicotinoid imidacloprid. *Bombus impatiens* is an increasingly important pollinator in US commercial agriculture^41^,^42^,^43^,^44^, and wild populations are common across eastern North America^45^. We provide evidence of variation in AMP expression in bumblebees in response to chemical exposure. In field-enclosed colonies, imidacloprid exposure significantly affected transcript levels for three of the four AMPs examined. Surprisingly, the direction of change was an increase in AMP expression following exposure.

## Results

### Variation in AMP expression in different body regions

**Figure 1:**
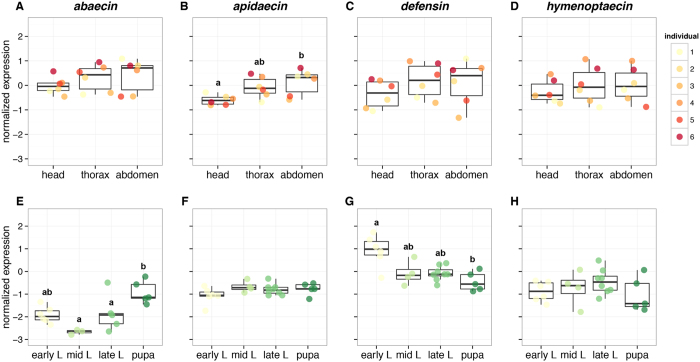
Expression of antimicrobial peptide genes encoding *abaecin, apidaecin, defensin* and *hymenoptaecin* from different body regions (**A**–**D**) and developmental stages (**E**–**H**) of *Bombus impatiens* raised in untreated, field enclosed colonies. The range of expression is indicated by Tukey’s plot: boxes demarcate the upper and lower quartiles, while the heavy bar indicates the median of normalized expression. Whiskers extend to 1.5 times the interquartile range or the most extreme value. Group differences according to post-hoc tests are indicated by different letters appearing above the whiskers for each group. Panels without letters indicate that no significant differences were found. Six individuals were used to examine expression by body region, and color-coding consistently indicates the same individual. Samples sizes for brood of each stage were 6 early larvae, 4 mid-stage larvae, 8 late larvae, and 5 pupae.

In order to determine the optimal sampling method, we examined AMP expression in different body regions of bees collected from separate colonies in field enclosures prior to pesticide treatment (Fig. 1A–D). Normalized AMP expression in the thorax and abdomen was similar in magnitude to samples from untreated whole foragers. However, expression of *apidaecin* was significantly lower in heads compared to abdomens (ANOVA *F*_2,15_ = 5.03, *p* = 0.0213; Tukey’s test head-abdomen *p* = 0.0197), with marginally less variance. Because of this difference, all other sampling was made using homogenates of whole adult bodies.

### Developmental time course of AMP expression

Two AMPs, *apidaecin* and *hymenoptaecin*, did not vary in their normalized expression among brood of different stages (Fig. 1F and H). An increase in normalized expression over development was detected for *abaecin* (Fig. 1E), with the lowest expression in mid-stage larvae and significantly higher normalized expression in pupae (Kruskal-Wallis rank sum test *χ*^2^_3_ = 12.129, *p* = 0.00695; Dunn’s test, pupae vs. mid-stage larvae *p* = 0.0026, late stage larvae *p* = 0.0355). In contrast, normalized expression of *defensin* (Fig. 1H) was highest in early larvae and decreased in older stages, with significantly lower expression in pupae (Kruskal-Wallis rank sum test *χ*^2^_3_ = 9.2955, *p* = 0.0256; Dunn’s test, early larvae vs. pupae *p* = 0.0099). These results show that AMPs in *B. impatiens* are subject to independent regulation in some biological contexts.

### AMP expression increased after imidacloprid exposure

Expression of four antimicrobial peptide genes was measured in bumblebees collected from colonies treated with a range of imidacloprid concentrations, administered in pollen and syrup. Concentrations were chosen to span the range of imidacloprid found in pollen and nectar of common crops after pesticide application (Table 1)^10^,^11^,^46^,^47^. In all experiments, hives that were not exposed to imidacloprid showed no significant change in mean AMP expression over time (Table 2). This includes colonies raised in the same location as previously treated colonies in the following year (Supplementary Fig. 1). However, significant correlations between the exposure time and the expression of AMPs were seen for all imidacloprid treatments (Table 2).

One possible confounding factor in this experiment is the possibility of infection. While it has not been demonstrate that infection induces AMP expression in *B. impatiens*, this has been shown in other insects^39^,^48^, including *B. terrestris*^36^. Examination of the abdominal interior did not reveal the presence of parasites in any of the sampled bees. Moreover, 18 individuals collected at day 19 from the control and high-concentration treatments were screened using realtime RT-PCR for the presence of *Nosema bombi* and *Crithidia bombi*, two of the most common pathogens of *B. impatiens*. No infections were identified (Supplementary Dataset S1).

**Figure 2:**
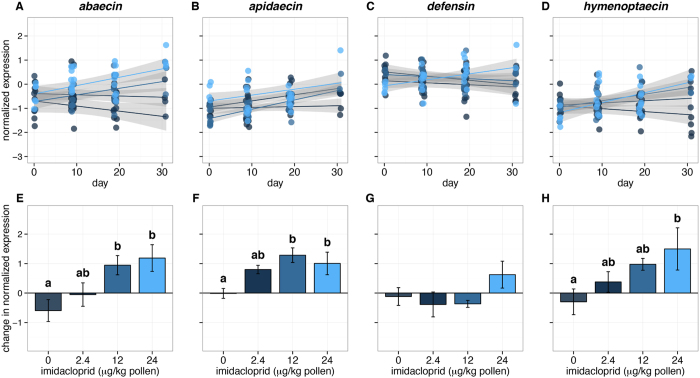
Expression of antimicrobial peptide genes from *B. impatiens* in captive colonies under differing imidacloprid dosage. Panels A–D show log_10_ expression over time, normalized against *actin-5C*. Dots represent individual samples. For each treatment group, a linear regression to the mean for each time point is shown with the gray area indicating the 95% confidence interval. Panels E-H show changes in the normalized log_10_ expression of each gene, from the first to last time points (+/− standard error). Treatments are shaded by dosage, consistently in both the top and bottom panels. For *abaecin, apidaecin* and *hymenoptaecin*, the effect of dosage was significant according to the Kruskal-Wallis rank sum test. Different letters indicate significant pairwise differences identified by Dunn’s test with Bonferroni correction. Sample sizes for most treatments at each time point are 3 individuals from 3 colonies for 9 total individuals. Exceptions include low dose, time zero, with n = 8; low dose, day 19, n = 7; control, day 31, n = 6 (from 2 colonies); low dose, day 31, n = 6; medium and high doses, day 31, n = 5.

Colonies in field enclosures that were treated with imidacloprid showed dose-dependent increases in normalized AMP expression over time (Fig. 2A,B and D). Significant dose-dependent positive correlations (Table 3) were found for the expression of *abaecin* after 9 days of exposure and for the duration of the experiment thereafter. Correlations to imidacloprid dose for expression of *apidaecin* and *hymenoptaecin* were also significant by day 31.

After 31 days of exposure to imidacloprid, expression differed significantly for *abaecin, apidaecin* and *hymenoptaecin* (Fig. 2; Kruskal-Wallis test for *abaecin* χ^2^_3_ = 11.19, *p* = 0.0107; for *apidaecin* χ^2^_3_ = 12.28, *p* = 0.00648; for *hymenoptaecin* χ^2^_3_ = 8.300, *p* = 0.0402). However *defensin* expression in each of the treated groups was not significantly different from the control treatment (Fig. 2C and G). Colony-level change over time in the expression of *abaecin* and *apidaecin* was significantly greater for imidacloprid treatments, starting at the 12 μg/kg pollen dose (Fig. 2E,F; Dunn’s test *abaecin* control vs.12 μg/kg *p* = 0.0292, vs. 24 μg/kg *p* = 0.0159, *apidaecin* control vs.12 μg/kg *p* = 0.0028, vs. 24 μg/kg *p* = 0.0372). Expression change in *hymenoptaecin* was significantly greater than controls only in the highest concentration treatment, 24 μg/kg (Fig. 2H; Dunn’s test *p* = 0.0176). The final masses of treated and control colonies, including all bees, brood, comb, and stored products, were not significantly different among treatments.

A replicate of the experiment was conducted the following year, in the same locations, without imidacloprid exposure (Fig. S1). In this mock experiment, no significant changes over time were detected for *abaecin, apidaecin* and *hymenoptaecin*. However, two mock-treatment blocks has significant differences in their change in *defensin* expression over the course of the experiment (Kruskal-Wallis *χ*^2^_3_ = 9.9038, *p* = 0.0194; Dunn’s test, blocks B–C *p* = 0.0325). Nevertheless, we interpret the outcome of this replicate to support the conclusion that changes in AMP expression in the first experiment resulted from imidacloprid exposure rather than confounding factors.

### Expression of AMPs in wild B. impatiens

**Figure 3:**
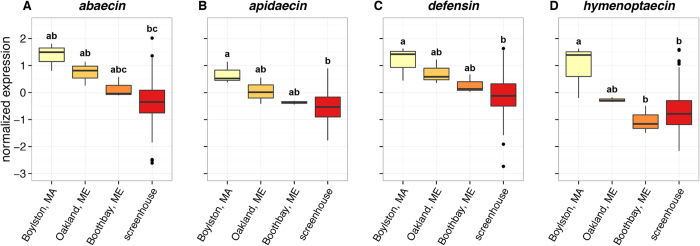
Expression of antimicrobial peptide genes in wild and captive *B. impatiens*, represented by Tukey’s plots. Expression is shown on a log_10_ scale, normalized against *actin-5c*. Wild bees were collected from field sites in Boylston, Massachusetts (n = 3), Oakland, Maine (n = 3), and Boothbay, Maine (n = 3). Samples were also taken from untreated colonies in experimental screen houses (n = 161) in Waterville, Maine. For each gene, the Kruskal-Wallis rank sum test identified locality as a significant factor Different letters indicate significant pairwise differences as determined by Dunn’s test with Bonferroni correction.

To compare the AMP expression levels in captive *B. impatiens* to those in wild populations (Fig. 3), we sampled foraging *B. impatiens* workers from pasture 3 km from the site of our field enclosures (Oakland, Maine), and at two botanical gardens, on the Atlantic coast (Boothbay, Maine) and inland (Boylston, Massachusetts). For all AMPs examined, a group consisting of all untreated bees from captive screen houses was not significantly different from wild *B. impatiens* collected at the two sites in Maine. However, bees collected from Boylston had significantly higher normalized expression of all AMPs than captive bees (Fig. 3). Expression of *hymenoptaecin* was also significantly higher in bees from Boylston compared to those collected from Boothbay. While Oakland was the closest site to the experimental screen houses, means of normalized expression for all AMPs were closer to values from Boothbay. It is unclear what local environmental factors might influence differences in AMP expression among collection sites. Expression of all 4 AMPs in wild *B. impatiens* was significantly correlated with the longitude of the collection site (Spearman’s rank correlation, *abaecin ρ* = −0.791, *p* = 0.0112; *apidaecin ρ* = −0.738, *p* = 0.0232; *defensin ρ* = −0.632, *p* = 0. 0.0676; *hymenoptaecin ρ* = −0.896, *p* = 0.00108). It is unclear what the proximate causes of this variation may be. However, it is possible that expression differences arise from variations in local pesticide exposure.

## Discussion

Here we report the first evidence that neonicotinoid exposure can alter the expression of antimicrobial peptide genes in bumblebees. It has been suggested that neonicotinoids may contribute to pollinator declines by increasing susceptibility to disease^6^,^7^,^31^. However, no physiological mechanism for this ecological pattern has been identified. One potential mechanism for a synergy of neonicotinoids and bee disease is the suppression of genes functioning in immunity after exposure to pesticide^29^. Published data on antimicrobial peptide expression in honeybees after neonicotinoid exposure have been ambiguous. One study reported reduced *abaecin* expression after clothianidin treatment^29^, while another found *hymenoptaecin*, alone among AMPs, was reduced in a treatment including imidacloprid exposure and *Nosema ceranae* infection^40^. In contrast, a study by Gregorc and colleagues^38^ exposed honeybees to a high imidacloprid concentration, but detected no changes in AMP expression. Based on these reports, we predicted AMP expression in *B. impatiens* might be reduced after chronic exposure to imidacloprid.

Contrary to these predictions, imidacloprid exposure in this study was associated with increased expression for three of the four AMP-encoding genes that were measured in a time- and dose-dependent manner (Fig. 2; Tables 2, 3). While the strongest effects were seen in the highest concentration treatments, these concentrations are within field-realistic levels for some crops, such as squash^46^,^47^. There are several possible interpretations of these results.

An increase in AMP expression at the doses used in this study may represent the excitatory phase of a hormetic or biphasic reaction norm^49^,^50^. If this is true, then similar experiments using higher concentrations of imidacloprid are predicted to eventually lead to decreased expression of AMP-encoding genes. Our study used concentrations of imidacloprid in order to approximate the conditions of chronic exposure associated with crops having relatively high systemic imidacloprid^46^,^47^. A hormetic response of innate immunity to neonicotinoids would significantly complicate the interactions of pollinators, pathogens and pesticides.

Another possible explanation for increased AMP expression is as a general physiological response to stress induced by the pesticide. For example, in one study of immunity-related gene expression, honeybees injected with saline buffer had similar increases in the expression of *AMPs* and other genes as those challenged with *E. coli* or *Paenibacillus larvae*^51^. However, while that study found correlated changes in the transcript levels of all sampled *AMPs* as well as other genes, the fact that change was not observed for *defensin* in our study suggests that effects are more direct and not a generalized up-regulation of immunity. In the future, it will be possible to use high-throughput sequencing to examine pesticide-induced changes in gene expression more broadly.

A direct effect on AMP expression is also supported by the fact that hive masses were not significantly different after imidacloprid exposure in our experiments. A previous study of *B. terrestris* reported significantly less growth in colonies dosed with imidacloprid^23^. However, that experiment dosed colonies in lab before allowing bees to freely forage. Our experiments limited feeding to screen houses in order to provide consistent chronic exposure. Since neonicotinoid exposure is known to impair foraging effectiveness^20^,^27^, higher growth in captivity might be explained by lower physical and perceptual demands on bees obtaining food compared to those freely foraging.

Finally, imidacloprid-induced increase in AMP expression may be unique to this species of bumblebee. While many *Bombus* species around the world have undergone population declines^4^,^7^, *B. impatiens* populations in North America have remained stable and abundant^45^. Therefore, it is possible that this species is atypical in its response, compared to other native pollinators or to *B. terrestris*, a European native that has served as a model social insect and pollinator.

Our data demonstrate that neonicotinoids can be modulators of immune gene expression in *B. impatiens* and suggest that local environmental factors, such as pesticide exposure, may produce significant differences in immune activity of *B. impatiens*. In the northeast United States, *B. impatiens* and other native bumblebees are crucial wild pollinators of regional crops, such as blueberry^41^, cranberry^42^ and raspberry^43^. More ecotoxicological research with *B. impatiens* is needed to better understand how this species might fare in the future in environments that are wild, agricultural or otherwise synanthropic.

## Methods

### Bumblebee colonies

Colonies of *Bombus impatiens* were obtained from Koppert Biological Systems, Inc. (Howell, Michigan, USA). In May of 2014 and 2015, colonies were started with one queen and 30 workers, randomly divided between treatment groups. All colonies were housed in a 6-L plastic mesh cage, with a gated entrance, covered in cardboard. To provide experimental conditions approximating the conditions of wild bees, experimental colonies were allowed limited foraging. Colonies were enclosed in screen houses covering 20-m^2^ of previously mown marginal meadowland in Waterville, Maine, USA. Flowering plants were removed from within the enclosures to ensure that bees consumed the food provided during the experiment. Bees were provided with honeybee pollen (YS Eco Bee Farms, Sheridan, Illinois, USA) homogenized in a dry rotary homogenizer (Proctor Silex, Glen Allen, Virginia, USA) for at least 1 minute. Pollen and 60% (v/v) cane sugar syrup were available at wooden feeding stations roughly 1 m from hive entrances, at a height of 1 m. All food was replaced when empty or after rain. Native bumblebees, including *B. impatiens, B. ternarius*, and *B. vagans*, normally use the study area for foraging and nesting. However, no native colonies were allowed in the area during the study. Hive masses were measured upon completion of the experiment on a portable balance as the weight of all bees, brood, comb, honey, pollen and other hive products, as well as the weight of the plastic cage.

### Experimental design

Imidacloprid of analytical grade (>98% purity; Sigma-Aldrich, St. Louis, Missouri, USA) was administered through pollen and syrup. Using distilled water, a 28 mg/L stock solution of imidacloprid was prepared using quantitative transfer. This stock was used to prepare syrup to the intended dosages. Pollen doses were prepared by combining powdered imidacloprid and homogenized pollen in a dry rotary homogenizer. Series dilutions (mass/mass) were used to obtain the treatment dosages.

Colonies were allowed to acclimate *in situ* for one week, before sampling or pesticide dosage. Starting May 2014, experimental colonies were distributed randomly among a vehicle control group and three imidacloprid dosage treatments. In each treatment, three colonies were provided untreated food or pollen and sugar syrup containing imidacloprid at low, medium or high doses (Table 1). These concentrations were chosen to reflect a realistic range that might be encountered by bees in the nectar and pollen on common crop plants^10^,^11^,^46^,^47^ and to correspond to similar, previous experiments of *B. terrestris* toxicology^23^.

In order to test whether trends observed in AMP expression were artifacts produced by the location of colonies in each treatment, the study design was replicated in 2015. Another set of colonies supplied by the same vendor was placed in the same locations and screen houses. All colonies received undosed food during this season, and samples were collected to examine variation over time.

### Sampling

Sampling was performed with colony- and individual-level replicates. Periodically, individuals were collected in triplicate from each colony. Collection targeted actively foraging bees. Therefore, individuals were collected emerging from or returning to nest boxes. Small emerging bees, which could potentially have been nurse bees, were avoided. Bees were collected during mid-day in 45-ml Corning snap-cap vials and anesthetized using FlyNap, a vapor of 50% triethylamine and 25% ethanol (Carolina Biological Supply Company, Burlington, North Carolina, USA). Individuals were then immediately decapitated, bisected and placed into RNAlater RNA Stabilization Reagent (Qiagen, Hilden, Germany). Within 1–4 hours, samples were transported to the laboratory and placed at −80 °C prior to RNA extraction.

In order to determine whether captive, commercially supplied bees were representative of wild *B. impatiens*, we also collected wild foraging *B. impatiens* from two sites in Maine and Massachusetts during June and July 2015. Mown, disused pastureland in Oakland, Maine, 3 km from the site of our field enclosures, was used as the most immediate comparison. Bumblebees were also collected from two botanical gardens, one on the Atlantic coast (Boothbay, Maine) and one roughly 50 km inland (Boylston, Massachusetts). At each site, three foraging *B. impatiens* was collected and stored in RNAlater as described above.

To determine an optimal sampling strategy for AMP gene expression measurement, additional bees collected from captive colonies were partitioned into head, thoracic (including legs and wings) and abdominal regions. Brood was also sampled from undosed colonies at the conclusion of field experiments in 2015. Brood was sorted by developmental stage and immediately used in RNA extraction.

### Quantification of Gene Expression

Expression of AMPs was measured using two-step quantitative real time PCR with multiplexed dual-labeled probes. Tissues were moved from RNAlater to a saline buffer containing β-mercaptoethanol and homogenized. Crude homogenate was purified by twice centrifuging at 20,000 × *g* for five minutes. This step was helpful in removing hard material, pile, and fats. In 2014, total RNA was extracted from supernatants using the PureLink RNA Mini Kit (Life Technologies, Carlsbad, California, USA). Samples collected in 2015 were processed using the Maxwell 16 LEV simplyRNA Tissue Kit (Promega, Madison, Wisconsin, USA). Template cDNA was produced by reverse transcription of 1 μg total RNA with an oligo-dT primer (iScript Select cDNA Synthesis Kit, BioRad, Hercules, California, USA).

For each target gene, primers and probes (Table 4) were designed using the Primer3 algorithm^52^. The mRNA sequences of *abaecin, apidaecin, defensin, hymenoptaecin* and *actin-5C* from *Bombus impatiens* were obtained from GenBank (accession numbers XM_003491496, XM_003491720, XM_003486302, XM_003494885 and XM_003488437, respectively). The specificity of products was verified by dissociation curves as well as sub-cloning and Sanger sequencing. Dual-labeled probes were synthesized by Sigma-Aldrich Custom Products (St. Louis, Missouri).

To produce quantitative template standards, plasmid clones of the target sequences were diluted to concentrations of 10^4^, 10^5^, 10^6^, and 10^7^ template molecules per μl^53^. Salmon sperm DNA (Life Technologies) was added to all standards at 50 ng/μl to approximate the milieu of non-specific sequences in actual cDNA samples. All primer-probe sets performed with high efficiency. Starting numbers of target transcripts were calculated from the mean of technical triplicates, based on the linear regression of standard log_10_-concentrations. Individual and colony replicates were kept separate for analysis. Data were saved in CSV format and imported for further analysis using R. Data files and the analysis script have been archived with the Dryad Digital Repository (doi:10.5061/dryad.3600k).

### Pathogen screening

Infection presents the possibility of a confounding factor in experimental analysis of AMP expression. Therefore, individual abdomens were visually inspected for parasites prior to homogenization. No macroendoparasites or mites were observed. We also screened 18 individuals from the control and high imidacloprid treatments from the day 19 collection, using realtime RT-PCR. The microsporidian *Nosema bombi* and the trypanosome *Crithidia bombi* are two of the most common pathogens of *B. impatiens*. Primers for each parasite were designed to highly expressed house-keeping genes. For *N. bombi*, methionine aminopeptidase (consensus of GenBank Accessions KF188772–KF188782 and JQ927011) was targeted using the primers Nb’map-161F, CGTCTAAAGAAGCTACGAATGCTG, and Nb’map-257R, TAGCTTCGCATTACTTCGTGGATA. *Crithidia bombi* was targeting by primers to GAPDH (GenBank Accession GU321192): Cb’gapdh-52F, GCGTACCAGATGAAGTTTGATACG, and Cb’gapdh-147R, AAGCACATCCGGCTTCTTCA. Long primers, spanning these gene sequences, were used to produce cloned gene fragments, for use as standards and positive amplification controls. These primers were parasite-specific and did not amplify from *B. impatiens* cDNAs known to be parasite-free. Realtime PCR was then used to test *B. impatiens* cDNAs from our experiment. Strong amplification was observed from positive controls containing parasite sequence. All amplicons were examined for dissociation curves that could confirm the amplification of parasite sequences. None of the 18 samples showed consistent parasite-specific amplification. Therefore, we attribute group differences in AMP expression to imidacloprid exposure, rather than to disease in individual bees.

### Statistical analysis

Statistical analyses and their output are presented in their entirety in the Supplementary Methods. Here we provide an overall description of statistical methods. All statistical tests and graphs were rendered using R, version 3.2.2^54^. Group comparisons were made using ANOVA or the Kruskal-Wallis rank sum test after tests of normality and homogeneity of variance. Post-hoc tests for pairwise comparisons were either Tukey’s honest significant difference, following ANOVA, or Dunn’s test with Bonferroni correction. For tests of correlation, we used Spearman’s rank correlation, which does not assume normality and is more robust to small sample sizes. Bonferroni correction was used to adjust *p*-values when multiple tests were performed on the same data. The log-transcript numbers of each AMP were normalized by subtraction of the value for *actin-5c* from within the same multiplex reaction. Change in normalized AMP expression over time was calculated by subtracting values for each individual at the final sample time point from the colony mean at the initial time point (Fig. 2E–H).

## Additional Information

**How to cite this article:** Simmons, W. R. and Angelini, D. R. Chronic exposure to a neonicotinoid increases expression of antimicrobial peptide genes in the bumblebee *Bombus impatiens. Sci. Rep.* **7**, 44773; doi: 10.1038/srep44773 (2017).

**Publisher's note:** Springer Nature remains neutral with regard to jurisdictional claims in published maps and institutional affiliations.

## Supplementary Material

Supplementary Information

## Figures and Tables

**Table 1:** Imidacloprid doses used in each treatment.

Treatment group	Imidacloprid (μg/kg)	
	Pollen	Syrup
control	0	0
low	2.4	0.28
medium	12	1.4
high	24	2.8

**Table 2:** Time-dependency of imidacloprid effects on AMP expression.

AMP	0 μg/kg		2.4 μg/kg		12 μg/kg		24 μg/kg	
	*ρ*	*p*	*ρ*	*p*	*ρ*	*p*	*ρ*	*p*
*abaecin*	−0.187	0.2981	−0.176	0.3520	0.404	0.0217*	0.513	0.002698**
*apidaecin*	0.104	0.5656	0.689	2.57 × 10^−5^***	0.684	1.58 × 10^−5^***	0.207	0.2554
*defensin*	−0.130	0.4719	−0.400	0.0285*	−0.260	0.1508	0.561	0.000847***
*hymenoptaecin*	−0.228	0.2014	0.074	0.6962	0.362	0.0417*	0.530	0.001819**

Spearman’s rank correlation *ρ* for normalized AMP expression for each imidacloprid dose. The listed concentrations are from the pollen supplied to foragers. *p < 0.05. **p < 0.01. ***p < 0.001.

**Table 3:** Dose-dependency of AMP expression.

AMP	day 0		day 9		day 19		day 31	
	*ρ*	*p*	*ρ*	*p*	*ρ*	*p*	*ρ*	*p*
*abaecin*	0.134	0.4431	0.483	0.002839**	0.400	0.0191*	0.759	4.29 × 10^−5^***
*apidaecin*	0.259	0.1323	0.256	0.1319	0.023	0.8991	0.821	2.86 × 10^−6^***
*defensin*	−0.102	0.5611	−0.012	0.9448	0.252	0.1497	0.405	0.0614
*hymenoptaecin*	−0.273	0.1121	0.237	0.1644	0.255	0.1450	0.616	0.00229**

Spearman’s rank correlation, *ρ*, is reported for normalized AMP expression with imidacloprid dose at each sampled time point.

**Table 4:** Oligonucleotide primers and dual-labeled probes used to measure AMP expression through multiplex qRT-PCR.

*gene*	oligo name	Sequence	amplicon	labels	
accession			(bp)	reporter	quencher
*actin 5 C*	Bi’act5C-1029P	TTCTGGTGACGGTGTTTCCC		Hex	BHQ-1
XM_003488437	Bi’act5C-986F	TTTCGCTATATGCTTCTGGACGTA	92		
	Bi’act5C-1077R	AGCGTATCCTTCGTAGATTGGTAC			
*abaecin*	Bi’aba-171P	AACCGTTTCCAAGCTTCCCA		FAM	BHQ-1
XM_003491496	Bi’aba-134F	TTTGTACCATATAATCCGCCACGA	103		
	Bi’aba-236R	GTAATGGGTATGGCCACTGAATTT			
*apidaecin*	Bi’apd-131P	AAAACTGAGCTCCGTCGTCG		Cy5.5	BHQ-3
XM_003491720	Bi’apd-104F	TCTACCACCACAATCCCAAATACA	114		
	Bi’apd-217R	AATTGGTGGGAGACTTATAGGTCG			
*defensin*	Bi’def-349P	CGAGAACGGAGTCTGCCTTT		Cy5	BHQ-3
XM_003486302	Bi’def-278F	ATCAAAGGAGTCGCTGAACATAGT	120		
	Bi’def-397R	CCAGAGATCCTTGAAGTTGGTCTT			
*hymenoptaecin*	Bi’hym-124P	GACAGAAACGGAGTGAACGC		Texas Red	BHQ-2
XM_003494885	Bi’hym-93F	CTTGGACGTCGATTATCATCAACG	81		
	Bi’hym-173R	GGACGAATATTCAGTCCACCGTAA			
