# Supplementary material for: Chronic exposure to a neonicotinoid increases expression of antimicrobial peptide genes in the bumblebee Bombus impatiens
Source: Sci Rep. 2017 Mar 21;7:44773. doi: 10.1038/srep44773 (PMC5359568; doi:10.1038/srep44773)
Supplement: Supplementary Information [file srep44773-s1.pdf]

Supplementary Information

for

Chronic exposure to a neonicotinoid increases expression of antimicrobial peptide genes in the bumblebee *Bombus impatiens*

William R. Simmons and David R. Angelini

correspondence to: david.r.angelini@gmail.com

This PDF file includes:

Supplementary Figure S1
Supplementary Methods

Other files associated with this manuscript:

Additional files related to this study are available from the Dryad Digital Repository at http://dx.doi.org/10.5061/dryad.3600k

Filename	Description
Bi.AMP.analysis.Rmd	Analysis script in R markdown, which was used to generate the Supplementary Methods
Bi.body.regions.csv	Dataset containing AMP gene expression measurements (as $\log_{10}$ transcripts / ng total RNA) for different *B. impatiens* body regions.
Bi.brood.csv	Dataset with gene expression measurements from brood of different stages
Bi.hive.masses.csv	Dataset of colony masses
Bi.imidacloprid.treatment.timecourse.csv	Dataset with gene expression measurements over times for the imidacloprid treated bees
Bi.mock.treatment.timecourse.csv	Dataset with gene expression measurements over times for a mock replicate experiment with no pesticide exposure
Bi.wild.csv	Dataset with gene expression measurements for wild-caught *B. impatiens*
supplementary.dataset.S1.xls	Supplementary Dataset S1, containing results from PCR-based pathogen screening of a subset of individuals from the imidacloprid treatments

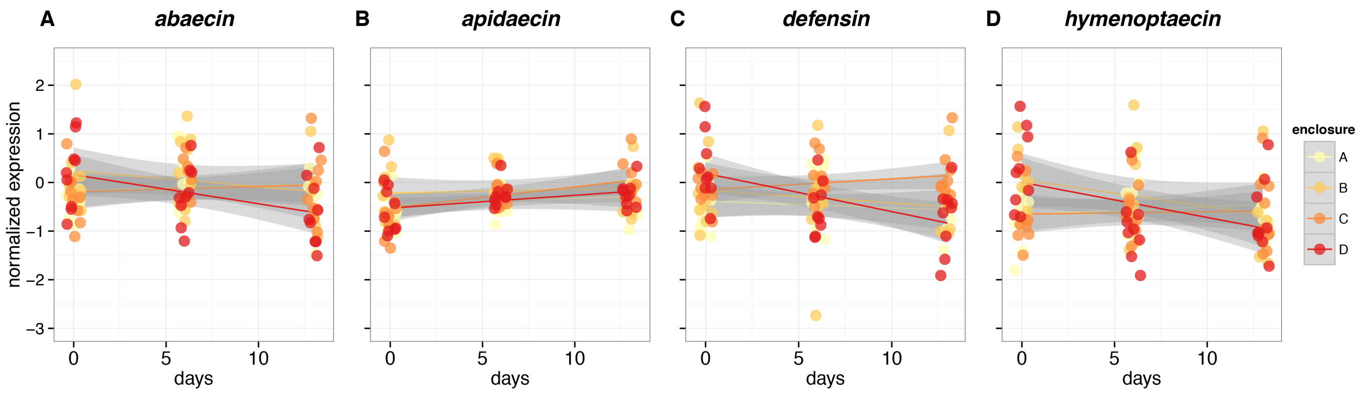

Supplementary Figure S1. Normalized expression of AMP-encoding genes in field enclosure without pesticide treatment. The same screen house enclosures used in the first experiment were used again for a second cohort of colonies without imidacloprid treatment. Sample sizes represent 3 individuals from 3 colonies in 4 enclosures (12 colonies total). Six individuals were not collected from the last time point, making the overall sample size for each time point 36, 36, and 30.

Supplementary Methods

for 'Chronic exposure to a neonicotinoid increases expression of antimicrobial peptide genes in the bumblebee *Bombus impatiens*'

William R. Simmons and David R. Angelini

This R Markdown document contains analyses from the study by Simmons and Angelini. This report was generated on 2017-01-14 using R version 3.3.2. All code and output from statistical analyses are shown. Code for the preparation of plots is not displayed in the PDF output, but they can be viewed in the original *Rmd* file. To repeat analyses and generation of plots, all the raw data files and scripts should be in the local folder.

AMP expression by body region

In order to determine the optimal sampling method, we examined AMP expression in different body regions of bees collected from separate colonies in field enclosures prior to pesticide treatment.

```
parts <- read.csv("Bi.body.regions.csv", header = T)
```

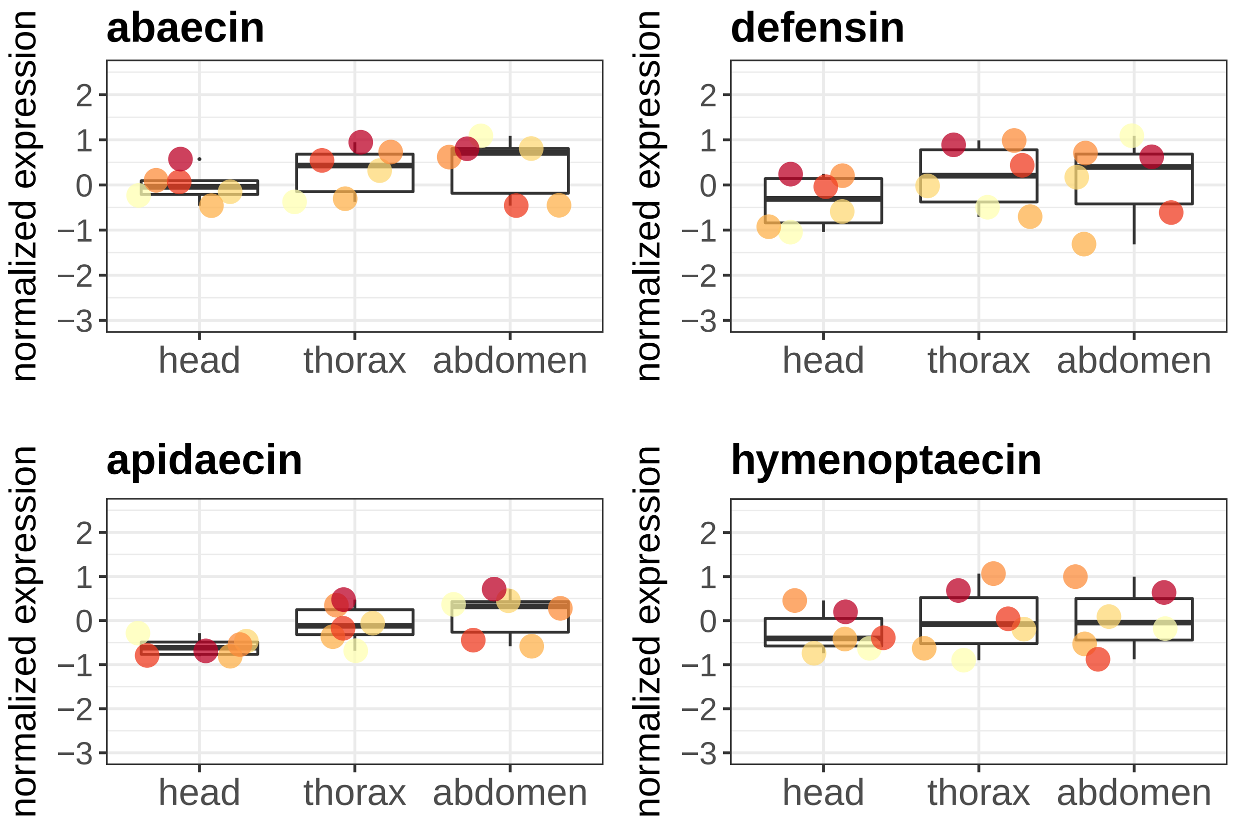

A version of this plot with all 4 panels in a row (using `cols=4`) was exported with as a PDF, at 4 x 15 inches (landscape), and appears in the manuscript as Figure 1A-D. Letters representing significant differences between groups (see below) were added in *Adobe Illustrator*.

Before preforming ANOVA, we tested assumptions using the Shapiro-Wilk test of normality and Levene's test for homogeneity of variance.

```
with(parts, by((aba-act5c),part,shapiro.test))
```

```
## part:   head
##
##  Shapiro-Wilk normality test
##
## data:  dd[x, ]
## W = 0.95937, p-value = 0.8148
##
## --------------------------------------------------------
## part:  thorax
##
##  Shapiro-Wilk normality test
##
## data:  dd[x, ]
## W = 0.9069, p-value = 0.4163
##
## --------------------------------------------------------
## part: abdomen
##
##  Shapiro-Wilk normality test
##
## data:  dd[x, ]
## W = 0.80496, p-value = 0.0651
```

```r
with(parts, by((apd-act5c),part,shapiro.test))
```

```
## part:   head
##
##  Shapiro-Wilk normality test
##
## data:  dd[x, ]
## W = 0.9327, p-value = 0.6011
##
## --------------------------------------------------------
## part:  thorax
##
##  Shapiro-Wilk normality test
##
## data:  dd[x, ]
## W = 0.96462, p-value = 0.8545
##
## --------------------------------------------------------
## part: abdomen
##
##  Shapiro-Wilk normality test
##
## data:  dd[x, ]
## W = 0.87793, p-value = 0.2597
```

```r
with(parts, by((def-act5c),part,shapiro.test))
```

```
## part:   head
##
##  Shapiro-Wilk normality test
##
## data:  dd[x, ]
```

```
## W = 0.87748, p-value = 0.2577
##
## --------------------------------------------------------
## part:  thorax
##
##  Shapiro-Wilk normality test
##
## data:  dd[x, ]
## W = 0.92024, p-value = 0.5071
##
## --------------------------------------------------------
## part: abdomen
##
##  Shapiro-Wilk normality test
##
## data:  dd[x, ]
## W = 0.92479, p-value = 0.5405
```

```
with(parts, by((hym-act5c),part,shapiro.test))
```

```
## part:   head
##
##  Shapiro-Wilk normality test
##
## data:  dd[x, ]
## W = 0.89026, p-value = 0.3196
##
## --------------------------------------------------------
## part:  thorax
##
##  Shapiro-Wilk normality test
##
## data:  dd[x, ]
## W = 0.95991, p-value = 0.819
##
## --------------------------------------------------------
## part: abdomen
##
##  Shapiro-Wilk normality test
##
## data:  dd[x, ]
## W = 0.97399, p-value = 0.9181
```

```
with(parts, leveneTest((aba-act5c),part))
```

```
## Levene's Test for Homogeneity of Variance (center = median)
##       Df F value Pr(>F)
## group  2  0.6292 0.5465
##       15
```

```
with(parts, leveneTest((apd-act5c),part))
```

```
## Levene's Test for Homogeneity of Variance (center = median)
##       Df F value Pr(>F)
## group  2   1.078 0.3652
##       15
```

```r
with(parts, leveneTest((def-act5c),part))
```

```
## Levene's Test for Homogeneity of Variance (center = median)
##       Df F value Pr(>F)
## group  2  0.4059 0.6735
##       15
```

```r
with(parts, leveneTest((hym-act5c),part))
```

```
## Levene's Test for Homogeneity of Variance (center = median)
##       Df F value Pr(>F)
## group  2   0.752 0.4884
##       15
```

The distributions of these data do not significantly deviate from normality or expectations of homogeneous variance. Therefore, we proceeded with tested for differences among body regions using ANOVA.

```r
summary(aov((aba-act5c)~part, data=parts))
```

```
##             Df Sum Sq Mean Sq F value Pr(>F)
## part         2  0.577  0.2885    0.98  0.398
## Residuals   15  4.417  0.2945
```

```r
summary(aov((apd-act5c)~part, data=parts))
```

```
##             Df Sum Sq Mean Sq F value Pr(>F)
## part         2  1.684  0.8419    5.03 0.0213 *
## Residuals   15  2.511  0.1674
## ---
## Signif. codes:  0 '***' 0.001 '**' 0.01 '*' 0.05 '.' 0.1 ' ' 1
```

```r
summary(aov((def-act5c)~part, data=parts))
```

```
##             Df Sum Sq Mean Sq F value Pr(>F)
## part         2  1.030  0.5149   0.933  0.415
## Residuals   15  8.275  0.5517
```

```r
summary(aov((hym-act5c)~part, data=parts))
```

```
##             Df Sum Sq Mean Sq F value Pr(>F)
## part         2  0.295  0.1475   0.342  0.716
## Residuals   15  6.471  0.4314
```

The expression of *apidaecin* differs among body regions. Tukey's Honest Significant Difference was used as the post-hoc test to identify pairwise differences.

```r
TukeyHSD(aov((apd-act5c)~part, data=parts))
```

```
##   Tukey multiple comparisons of means
##     95% family-wise confidence level
##
## Fit: aov(formula = (apd - act5c) ~ part, data = parts)
##
## $part
##                    diff         lwr       upr     p adj
##  thorax-  head  0.52000 -0.09353688 1.1335369 0.1033567
## abdomen-  head  0.72705  0.11351312 1.3405869 0.0197166
## abdomen- thorax 0.20705 -0.40648688 0.8205869 0.6627203
```

Developmental time course

We were interested in whether the expression of AMPs might vary by developmental stage.

```
brood <- read.csv("Bi.brood.csv", header = T)
as.table(with(brood, by(stage,stage,length)))
```

```
## stage
## early larva  late larva   mid larva        pupa
##           6           8           4           5
```

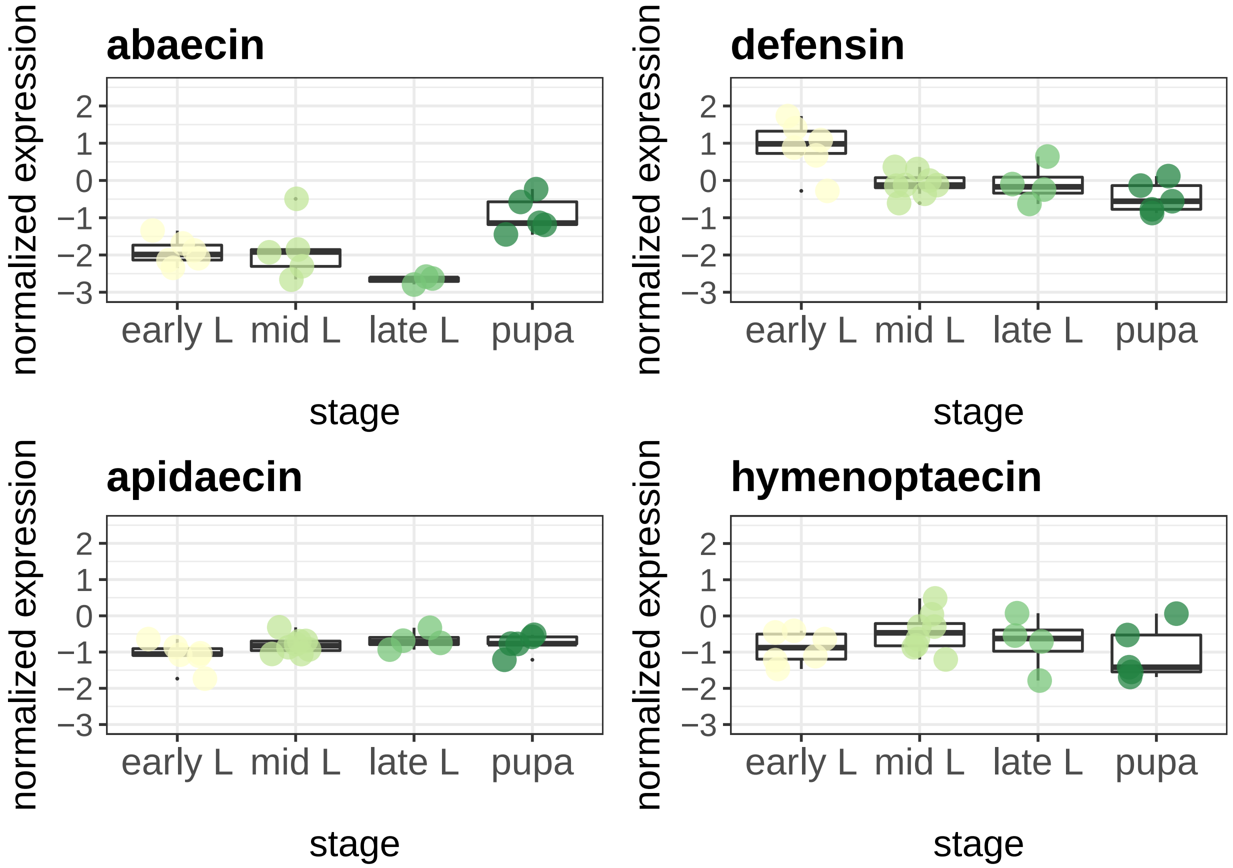

A version of this plot with all 4 panels in a row (using `cols=4`) was exported with as a PDF, at 4 x 15 inches (landscape), and appears in the manuscript as Figure 1E-H. Letters representing significant differences between groups (see below) were added in *Adobe Illustrator*.

We tested ANOVA assumptions using the Shapiro-Wilk test of normality and Levene's test for homogeneity of variance.

```
with(brood, by((aba-act5c),stage,shapiro.test))
```

```
## stage: early larva
##
##  Shapiro-Wilk normality test
##
## data:  dd[x, ]
## W = 0.96703, p-value = 0.8719
##
## -------------------------------------------------------------
## stage: late larva
```

```
##
##  Shapiro-Wilk normality test
##
## data:  dd[x, ]
## W = 0.97809, p-value = 0.9498
##
## ---------------------------------------------------------
## stage: mid larva
##
##  Shapiro-Wilk normality test
##
## data:  dd[x, ]
## W = 0.73957, p-value = 0.0307
##
## ---------------------------------------------------------
## stage: pupa
##
##  Shapiro-Wilk normality test
##
## data:  dd[x, ]
## W = 0.92277, p-value = 0.548
```

```
with(brood, by((apd-act5c),stage,shapiro.test))
```

```
## stage: early larva
##
##  Shapiro-Wilk normality test
##
## data:  dd[x, ]
## W = 0.89109, p-value = 0.324
##
## ---------------------------------------------------------
## stage: late larva
##
##  Shapiro-Wilk normality test
##
## data:  dd[x, ]
## W = 0.90429, p-value = 0.3156
##
## ---------------------------------------------------------
## stage: mid larva
##
##  Shapiro-Wilk normality test
##
## data:  dd[x, ]
## W = 0.9448, p-value = 0.6838
##
## ---------------------------------------------------------
## stage: pupa
##
##  Shapiro-Wilk normality test
##
## data:  dd[x, ]
## W = 0.8728, p-value = 0.2779
```

```r
with(brood, by((def-act5c),stage,shapiro.test))
```

```
## stage: early larva
##
##  Shapiro-Wilk normality test
##
## data:  dd[x, ]
## W = 0.94886, p-value = 0.7311
##
## --------------------------------------------------------
## stage: late larva
##
##  Shapiro-Wilk normality test
##
## data:  dd[x, ]
## W = 0.94555, p-value = 0.6664
##
## --------------------------------------------------------
## stage: mid larva
##
##  Shapiro-Wilk normality test
##
## data:  dd[x, ]
## W = 0.94882, p-value = 0.7087
##
## --------------------------------------------------------
## stage: pupa
##
##  Shapiro-Wilk normality test
##
## data:  dd[x, ]
## W = 0.92229, p-value = 0.5448
```

```r
with(brood, by((hym-act5c),stage,shapiro.test))
```

```
## stage: early larva
##
##  Shapiro-Wilk normality test
##
## data:  dd[x, ]
## W = 0.89967, p-value = 0.372
##
## --------------------------------------------------------
## stage: late larva
##
##  Shapiro-Wilk normality test
##
## data:  dd[x, ]
## W = 0.97142, p-value = 0.9089
##
## --------------------------------------------------------
## stage: mid larva
##
##  Shapiro-Wilk normality test
```

```
##
## data:  dd[x, ]
## W = 0.94843, p-value = 0.7063
##
## ---------------------------------------------------------
## stage: pupa
##
##  Shapiro-Wilk normality test
##
## data:  dd[x, ]
## W = 0.86495, p-value = 0.2466
```

```r
with(brood, leveneTest((aba-act5c),stage))
```

```
## Levene's Test for Homogeneity of Variance (center = median)
##       Df F value Pr(>F)
## group  3  1.3451 0.2912
##       18
```

```r
with(brood, leveneTest((apd-act5c),stage))
```

```
## Levene's Test for Homogeneity of Variance (center = median)
##       Df F value Pr(>F)
## group  3  0.1126 0.9516
##       19
```

```r
with(brood, leveneTest((def-act5c),stage))
```

```
## Levene's Test for Homogeneity of Variance (center = median)
##       Df F value Pr(>F)
## group  3  0.7987 0.5098
##       19
```

```r
with(brood, leveneTest((hym-act5c),stage))
```

```
## Levene's Test for Homogeneity of Variance (center = median)
##       Df F value Pr(>F)
## group  3  0.1968 0.8973
##       19
```

Data are not normally distributed for mid-stage larvae. Therefore comparisons were made with nonparametric tests.

```r
kruskal.test((aba-act5c)~stage, data=brood)
```

```
##
##  Kruskal-Wallis rank sum test
##
## data:  (aba - act5c) by stage
## Kruskal-Wallis chi-squared = 12.129, df = 3, p-value = 0.006953
```

```r
kruskal.test((apd-act5c)~stage, data=brood)
```

```
##
##  Kruskal-Wallis rank sum test
##
## data:  (apd - act5c) by stage
## Kruskal-Wallis chi-squared = 4.3098, df = 3, p-value = 0.2299
```

```r
kruskal.test((def-act5c)~stage, data=brood)
```

```
##
##  Kruskal-Wallis rank sum test
##
## data:  (def - act5c) by stage
## Kruskal-Wallis chi-squared = 9.2955, df = 3, p-value = 0.02561
```

```r
kruskal.test((hym-act5c)~stage, data=brood)
```

```
##
##  Kruskal-Wallis rank sum test
##
## data:  (hym - act5c) by stage
## Kruskal-Wallis chi-squared = 2.5832, df = 3, p-value = 0.4605
```

The expression of *abaecin* and *defensin* differ among brood stages. Dunn's test with Bonferroni correction was used to identify pairwise differences.

```r
with(brood, dunn.test((aba-act5c), g=stage, method="bonferroni"))
```

```
##   Kruskal-Wallis rank sum test
##
## data: x and stage
## Kruskal-Wallis chi-squared = 12.1293, df = 3, p-value = 0.01
##
##
##                            Comparison of x by stage
##                                  (Bonferroni)
## Col Mean-|
## Row Mean |   early la   late lar   mid larv
## ---------+---------------------------------
## late lar |   0.804042
##          |     1.0000
##          |
## mid larv |   1.868820   1.210930
##          |     0.1849     0.6778
##          |
##     pupa |  -1.695463  -2.517303  -3.328718
##          |     0.2700     0.0355     0.0026
```

```r
with(brood, dunn.test((def-act5c), g=stage, method="bonferroni"))
```

```
##   Kruskal-Wallis rank sum test
##
## data: x and stage
## Kruskal-Wallis chi-squared = 9.2955, df = 3, p-value = 0.03
##
##
##                            Comparison of x by stage
##                                  (Bonferroni)
## Col Mean-|
## Row Mean |   early la   late lar   mid larv
## ---------+---------------------------------
## late lar |   2.058946
##          |     0.1185
```

```
##           |
## mid larv |   1.865398   0.150482
##           |     0.1864     1.0000
##           |
##      pupa |   2.938141   1.170302   0.857194
##           |     0.0099     0.7256     1.0000
```

Imidacloprid treatment

```
imid <- read.csv("Bi.imidacloprid.treatment.timecourse.csv", header = T)
```

This dataset contains values for AMP expression (as $\log_{10}$ transcripts / ng total RNA), as well as values for the reference gene *actin-5c*. These values are based on the mean C_t of technical triplicate realtime PCR reactions compared by linear regression to a series of 4 concentration standards. The dataset includes samples from 127 individual bees. Samples were taken in 4 collections over a 31-day period. The experiment included 3 imidacloprid doses and a control treatment, each with 3 replicate colonies. At each collection time, 3 individual bees were sampled from each colony. We eliminated 17 samples which failed to produce RNA of suitable quantitity or quality.

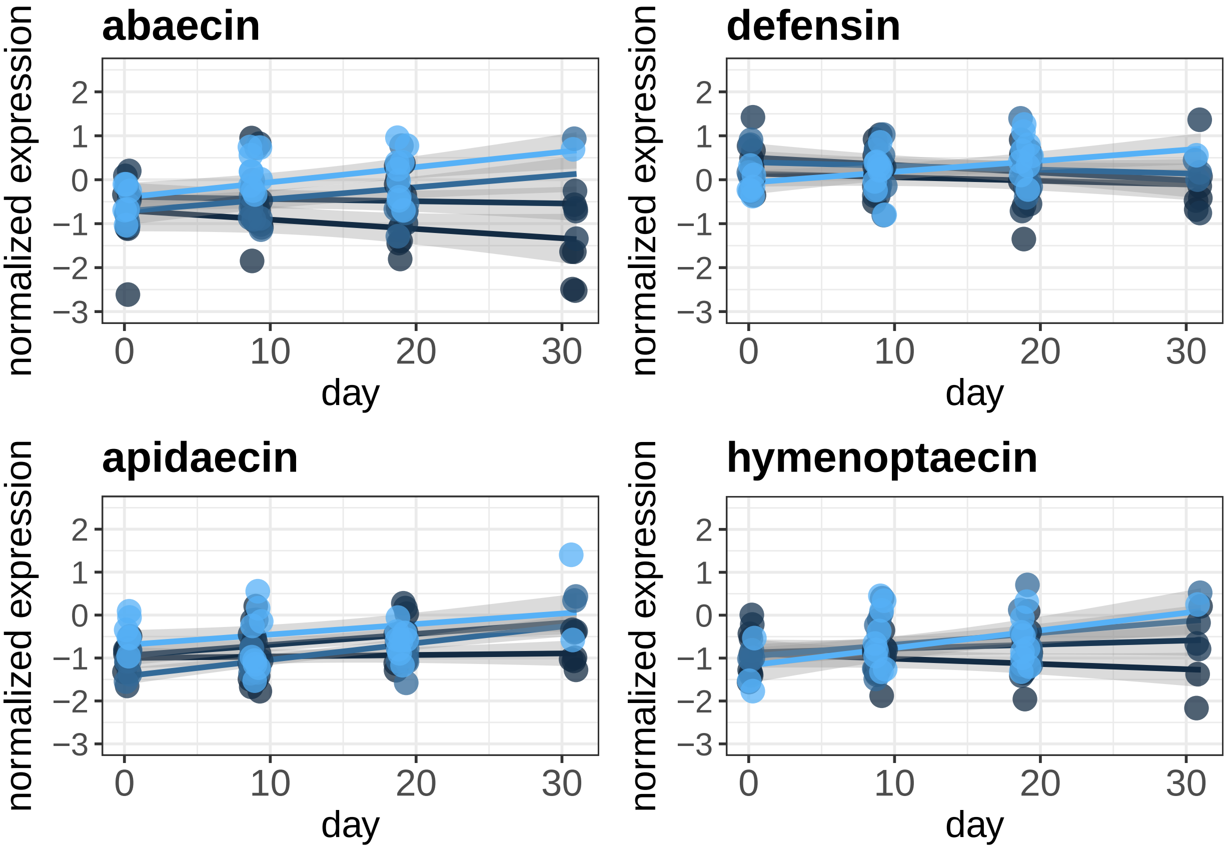

A version of this plot with all 4 panels in a row (using `cols=4`) was exported with as a PDF, at 4 x 15 inches (landscape), and appears in the manuscript as Figure 2A-D.

Time-dependency

We first tested for normality to determine the appropriate method to test for correlations (Pearson's or Spearman's) among measurements of AMP expression grouped by dosage and sampling time.

```r
dose_day <- as.factor(paste(paste(imid$pollen_dose,"ppb",sep=""),paste(imid$day,"d",sep=""),sep="/"))
with(imid, by((aba-act5c),dose_day,shapiro.test))
```

```
## dose_day: 0ppb/0d
##
##  Shapiro-Wilk normality test
##
## data:  dd[x, ]
## W = 0.92273, p-value = 0.4153
##
## --------------------------------------------------------
## dose_day: 0ppb/19d
##
##  Shapiro-Wilk normality test
##
## data:  dd[x, ]
## W = 0.93422, p-value = 0.5225
##
## --------------------------------------------------------
## dose_day: 0ppb/31d
##
##  Shapiro-Wilk normality test
##
## data:  dd[x, ]
## W = 0.82636, p-value = 0.1001
##
## --------------------------------------------------------
## dose_day: 0ppb/9d
##
##  Shapiro-Wilk normality test
##
## data:  dd[x, ]
## W = 0.87347, p-value = 0.1339
##
## --------------------------------------------------------
## dose_day: 12ppb/0d
##
##  Shapiro-Wilk normality test
##
## data:  dd[x, ]
## W = 0.9131, p-value = 0.3382
##
## --------------------------------------------------------
## dose_day: 12ppb/19d
##
##  Shapiro-Wilk normality test
##
## data:  dd[x, ]
## W = 0.90002, p-value = 0.2521
##
## --------------------------------------------------------
## dose_day: 12ppb/31d
##
##  Shapiro-Wilk normality test
```

```
##
## data:  dd[x, ]
## W = 0.74062, p-value = 0.02431
##
## ---------------------------------------------------------
## dose_day: 12ppb/9d
##
##  Shapiro-Wilk normality test
##
## data:  dd[x, ]
## W = 0.90575, p-value = 0.2872
##
## ---------------------------------------------------------
## dose_day: 2.4ppb/0d
##
##  Shapiro-Wilk normality test
##
## data:  dd[x, ]
## W = 0.89932, p-value = 0.2849
##
## ---------------------------------------------------------
## dose_day: 2.4ppb/19d
##
##  Shapiro-Wilk normality test
##
## data:  dd[x, ]
## W = 0.92222, p-value = 0.4868
##
## ---------------------------------------------------------
## dose_day: 2.4ppb/31d
##
##  Shapiro-Wilk normality test
##
## data:  dd[x, ]
## W = 0.98195, p-value = 0.9608
##
## ---------------------------------------------------------
## dose_day: 2.4ppb/9d
##
##  Shapiro-Wilk normality test
##
## data:  dd[x, ]
## W = 0.68575, p-value = 0.0009673
##
## ---------------------------------------------------------
## dose_day: 24ppb/0d
##
##  Shapiro-Wilk normality test
##
## data:  dd[x, ]
## W = 0.92701, p-value = 0.4534
##
## ---------------------------------------------------------
## dose_day: 24ppb/19d
```

```
##
##  Shapiro-Wilk normality test
##
## data:  dd[x, ]
## W = 0.95459, p-value = 0.7403
##
## --------------------------------------------------------
## dose_day: 24ppb/31d
##
##  Shapiro-Wilk normality test
##
## data:  dd[x, ]
## W = 0.88716, p-value = 0.343
##
## --------------------------------------------------------
## dose_day: 24ppb/9d
##
##  Shapiro-Wilk normality test
##
## data:  dd[x, ]
## W = 0.91448, p-value = 0.3485
```

```
with(imid, by((apd-act5c),dose_day,shapiro.test))
```

```
## dose_day: 0ppb/0d
##
##  Shapiro-Wilk normality test
##
## data:  dd[x, ]
## W = 0.94068, p-value = 0.5891
##
## --------------------------------------------------------
## dose_day: 0ppb/19d
##
##  Shapiro-Wilk normality test
##
## data:  dd[x, ]
## W = 0.96217, p-value = 0.8207
##
## --------------------------------------------------------
## dose_day: 0ppb/31d
##
##  Shapiro-Wilk normality test
##
## data:  dd[x, ]
## W = 0.79483, p-value = 0.05278
##
## --------------------------------------------------------
## dose_day: 0ppb/9d
##
##  Shapiro-Wilk normality test
##
## data:  dd[x, ]
## W = 0.81604, p-value = 0.03108
##
```

```
## ----------------------------------------------------------
## dose_day: 12ppb/0d
##
##  Shapiro-Wilk normality test
##
## data:  dd[x, ]
## W = 0.90541, p-value = 0.2851
##
## ----------------------------------------------------------
## dose_day: 12ppb/19d
##
##  Shapiro-Wilk normality test
##
## data:  dd[x, ]
## W = 0.9423, p-value = 0.6063
##
## ----------------------------------------------------------
## dose_day: 12ppb/31d
##
##  Shapiro-Wilk normality test
##
## data:  dd[x, ]
## W = 0.79797, p-value = 0.078
##
## ----------------------------------------------------------
## dose_day: 12ppb/9d
##
##  Shapiro-Wilk normality test
##
## data:  dd[x, ]
## W = 0.84552, p-value = 0.06651
##
## ----------------------------------------------------------
## dose_day: 2.4ppb/0d
##
##  Shapiro-Wilk normality test
##
## data:  dd[x, ]
## W = 0.92167, p-value = 0.4436
##
## ----------------------------------------------------------
## dose_day: 2.4ppb/19d
##
##  Shapiro-Wilk normality test
##
## data:  dd[x, ]
## W = 0.88998, p-value = 0.2745
##
## ----------------------------------------------------------
## dose_day: 2.4ppb/31d
##
##  Shapiro-Wilk normality test
##
## data:  dd[x, ]
```

```
## W = 0.70678, p-value = 0.007281
##
## ---------------------------------------------------------
## dose_day: 2.4ppb/9d
##
##  Shapiro-Wilk normality test
##
## data:  dd[x, ]
## W = 0.91522, p-value = 0.3542
##
## ---------------------------------------------------------
## dose_day: 24ppb/0d
##
##  Shapiro-Wilk normality test
##
## data:  dd[x, ]
## W = 0.98167, p-value = 0.9723
##
## ---------------------------------------------------------
## dose_day: 24ppb/19d
##
##  Shapiro-Wilk normality test
##
## data:  dd[x, ]
## W = 0.97435, p-value = 0.9292
##
## ---------------------------------------------------------
## dose_day: 24ppb/31d
##
##  Shapiro-Wilk normality test
##
## data:  dd[x, ]
## W = 0.93511, p-value = 0.6316
##
## ---------------------------------------------------------
## dose_day: 24ppb/9d
##
##  Shapiro-Wilk normality test
##
## data:  dd[x, ]
## W = 0.91895, p-value = 0.3836
```

```r
with(imid, by((def-act5c),dose_day,shapiro.test))
```

```
## dose_day: 0ppb/0d
##
##  Shapiro-Wilk normality test
##
## data:  dd[x, ]
## W = 0.9141, p-value = 0.3456
##
## ---------------------------------------------------------
## dose_day: 0ppb/19d
##
##  Shapiro-Wilk normality test
```

```
##
## data:  dd[x, ]
## W = 0.8931, p-value = 0.2146
##
## ------------------------------------------------------------
## dose_day: 0ppb/31d
##
##  Shapiro-Wilk normality test
##
## data:  dd[x, ]
## W = 0.82864, p-value = 0.1047
##
## ------------------------------------------------------------
## dose_day: 0ppb/9d
##
##  Shapiro-Wilk normality test
##
## data:  dd[x, ]
## W = 0.9791, p-value = 0.9595
##
## ------------------------------------------------------------
## dose_day: 12ppb/0d
##
##  Shapiro-Wilk normality test
##
## data:  dd[x, ]
## W = 0.95146, p-value = 0.7061
##
## ------------------------------------------------------------
## dose_day: 12ppb/19d
##
##  Shapiro-Wilk normality test
##
## data:  dd[x, ]
## W = 0.89842, p-value = 0.243
##
## ------------------------------------------------------------
## dose_day: 12ppb/31d
##
##  Shapiro-Wilk normality test
##
## data:  dd[x, ]
## W = 0.86633, p-value = 0.2519
##
## ------------------------------------------------------------
## dose_day: 12ppb/9d
##
##  Shapiro-Wilk normality test
##
## data:  dd[x, ]
## W = 0.94482, p-value = 0.6335
##
## ------------------------------------------------------------
## dose_day: 2.4ppb/0d
```

```
##
##  Shapiro-Wilk normality test
##
## data:  dd[x, ]
## W = 0.94161, p-value = 0.627
##
## --------------------------------------------------------
## dose_day: 2.4ppb/19d
##
##  Shapiro-Wilk normality test
##
## data:  dd[x, ]
## W = 0.96235, p-value = 0.8386
##
## --------------------------------------------------------
## dose_day: 2.4ppb/31d
##
##  Shapiro-Wilk normality test
##
## data:  dd[x, ]
## W = 0.98581, p-value = 0.9765
##
## --------------------------------------------------------
## dose_day: 2.4ppb/9d
##
##  Shapiro-Wilk normality test
##
## data:  dd[x, ]
## W = 0.93407, p-value = 0.5211
##
## --------------------------------------------------------
## dose_day: 24ppb/0d
##
##  Shapiro-Wilk normality test
##
## data:  dd[x, ]
## W = 0.92958, p-value = 0.4773
##
## --------------------------------------------------------
## dose_day: 24ppb/19d
##
##  Shapiro-Wilk normality test
##
## data:  dd[x, ]
## W = 0.98839, p-value = 0.9935
##
## --------------------------------------------------------
## dose_day: 24ppb/31d
##
##  Shapiro-Wilk normality test
##
## data:  dd[x, ]
## W = 0.89768, p-value = 0.3972
##
```

```
## ---------------------------------------------------------
## dose_day: 24ppb/9d
##
##  Shapiro-Wilk normality test
##
## data:  dd[x, ]
## W = 0.93361, p-value = 0.5164
```

```r
with(imid, by((hym-act5c),dose_day,shapiro.test))
```

```
## dose_day: 0ppb/0d
##
##  Shapiro-Wilk normality test
##
## data:  dd[x, ]
## W = 0.89191, p-value = 0.2087
##
## ---------------------------------------------------------
## dose_day: 0ppb/19d
##
##  Shapiro-Wilk normality test
##
## data:  dd[x, ]
## W = 0.96167, p-value = 0.8157
##
## ---------------------------------------------------------
## dose_day: 0ppb/31d
##
##  Shapiro-Wilk normality test
##
## data:  dd[x, ]
## W = 0.79966, p-value = 0.05836
##
## ---------------------------------------------------------
## dose_day: 0ppb/9d
##
##  Shapiro-Wilk normality test
##
## data:  dd[x, ]
## W = 0.91369, p-value = 0.3426
##
## ---------------------------------------------------------
## dose_day: 12ppb/0d
##
##  Shapiro-Wilk normality test
##
## data:  dd[x, ]
## W = 0.93619, p-value = 0.5424
##
## ---------------------------------------------------------
## dose_day: 12ppb/19d
##
##  Shapiro-Wilk normality test
##
## data:  dd[x, ]
```

```
## W = 0.81965, p-value = 0.03414
##
## ----------------------------------------------------------
## dose_day: 12ppb/31d
##
##  Shapiro-Wilk normality test
##
## data:  dd[x, ]
## W = 0.81678, p-value = 0.1102
##
## ----------------------------------------------------------
## dose_day: 12ppb/9d
##
##  Shapiro-Wilk normality test
##
## data:  dd[x, ]
## W = 0.91287, p-value = 0.3365
##
## ----------------------------------------------------------
## dose_day: 2.4ppb/0d
##
##  Shapiro-Wilk normality test
##
## data:  dd[x, ]
## W = 0.94406, p-value = 0.6514
##
## ----------------------------------------------------------
## dose_day: 2.4ppb/19d
##
##  Shapiro-Wilk normality test
##
## data:  dd[x, ]
## W = 0.93098, p-value = 0.5592
##
## ----------------------------------------------------------
## dose_day: 2.4ppb/31d
##
##  Shapiro-Wilk normality test
##
## data:  dd[x, ]
## W = 0.93997, p-value = 0.6589
##
## ----------------------------------------------------------
## dose_day: 2.4ppb/9d
##
##  Shapiro-Wilk normality test
##
## data:  dd[x, ]
## W = 0.78854, p-value = 0.0151
##
## ----------------------------------------------------------
## dose_day: 24ppb/0d
##
##  Shapiro-Wilk normality test
```

```
##
## data:  dd[x, ]
## W = 0.89175, p-value = 0.2079
##
## --------------------------------------------------------
## dose_day: 24ppb/19d
##
##  Shapiro-Wilk normality test
##
## data:  dd[x, ]
## W = 0.96058, p-value = 0.8044
##
## --------------------------------------------------------
## dose_day: 24ppb/31d
##
##  Shapiro-Wilk normality test
##
## data:  dd[x, ]
## W = 0.90139, p-value = 0.4176
##
## --------------------------------------------------------
## dose_day: 24ppb/9d
##
##  Shapiro-Wilk normality test
##
## data:  dd[x, ]
## W = 0.91997, p-value = 0.392
```

Data for several dose / day combinations are not normally distributed. Therefore, we tested for corrleation using Spearman's rank correlation.

```
amp.names <- c("aba","apd","def","hym")
dose.factor <- levels(as.factor(imid$pollen_dose))
time.rho <- data.frame(row.names = amp.names)
for (i in 1:length(dose.factor)) {
  x <- (i-1)*3+1
  d <- which(imid$pollen_dose==as.numeric(dose.factor[i]))
  s <- with(imid, cor.test(day[d],(aba[d]-act5c[d]), method = "spearman"))
  time.rho[1,x] <- round(s$estimate,3)
  time.rho[1,x+1] <- signif(s$p.value,4)
  time.rho[1,x+2]<- symnum(time.rho[1,x+1], corr = FALSE, cutpoints = c(0, 0.001, 0.01, 0.05, 0.1, 1), s
  s <- with(imid, cor.test(day[d],(apd[d]-act5c[d]), method = "spearman"))
  time.rho[2,x] <- round(s$estimate,3)
  time.rho[2,x+1] <- signif(s$p.value,4)
  time.rho[2,x+2]<- symnum(time.rho[2,x+1], corr = FALSE, cutpoints = c(0, 0.001, 0.01, 0.05, 0.1, 1), s
  s <- with(imid, cor.test(day[d],(def[d]-act5c[d]), method = "spearman"))
  time.rho[3,x] <- round(s$estimate,3)
  time.rho[3,x+1] <- signif(s$p.value,4)
  time.rho[3,x+2]<- symnum(time.rho[3,x+1], corr = FALSE, cutpoints = c(0, 0.001, 0.01, 0.05, 0.1, 1), s
  s <- with(imid, cor.test(day[d],(hym[d]-act5c[d]), method = "spearman"))
  time.rho[4,x] <- round(s$estimate,3)
  time.rho[4,x+1] <- signif(s$p.value,4)
  time.rho[4,x+2]<- symnum(time.rho[4,x+1], corr = FALSE, cutpoints = c(0, 0.001, 0.01, 0.05, 0.1, 1), s
}
dose.factor <- c(" 0ppb"," 2.4ppb","12ppb","24ppb")
```

```
x <- sort(c(paste(dose.factor,"rho"),paste(dose.factor,"p",sep="_")))
colnames(time.rho) <- c(x[1:2],"",x[3:4],"",x[5:6],"",x[7:8],"")
time.rho
```

```
##      0ppb rho  0ppb_p      2.4ppb rho  2.4ppb_p     12ppb rho   12ppb_p
## aba    -0.187  0.2981          -0.176 3.520e-01         0.404 2.171e-02
## apd     0.104  0.5656           0.689 2.568e-05 ***     0.684 1.583e-05
## def    -0.130  0.4719          -0.400 2.851e-02 *      -0.260 1.508e-01
## hym    -0.228  0.2014           0.074 6.962e-01         0.362 4.165e-02
##          24ppb rho  24ppb_p
## aba *       0.513 0.002698 **
## apd ***     0.207 0.255400
## def         0.561 0.000847 ***
## hym *       0.530 0.001819 **
```

The output above appears in the main text as Table 2.

In the control treatment, there was no correlation between the date of sampling and the expression of AMPs. However, significant correlations between the time since introduction of imidacloprid and the expression of several AMPs was seen for all three of the applied imidacloprid dosages.

Dose dependency

We considered whether the expression of each AMP correlated with imidacloprid dose at each day of the experiment.

```
days.factor <- levels(as.factor(imid$day))
dose.rho <- data.frame(row.names = amp.names)
for (i in 1:length(days.factor)) {
  x <- (i-1)*3+1
  d <- which(imid$day==as.numeric(days.factor[i]))
  s <- with(imid, cor.test(pollen_dose[d],(aba[d]-act5c[d]), method = "spearman"))
  dose.rho[1,x] <- round(s$estimate,3)
  dose.rho[1,x+1] <- signif(s$p.value,4)
  dose.rho[1,x+2]<- symnum(dose.rho[1,x+1], corr = FALSE, cutpoints = c(0, 0.001, 0.01, 0.05, 0.1, 1), s
  s <- with(imid, cor.test(pollen_dose[d],(apd[d]-act5c[d]), method = "spearman"))
  dose.rho[2,x] <- round(s$estimate,3)
  dose.rho[2,x+1] <- signif(s$p.value,4)
  dose.rho[2,x+2]<- symnum(dose.rho[2,x+1], corr = FALSE, cutpoints = c(0, 0.001, 0.01, 0.05, 0.1, 1), s
  s <- with(imid, cor.test(pollen_dose[d],(def[d]-act5c[d]), method = "spearman"))
  dose.rho[3,x] <- round(s$estimate,3)
  dose.rho[3,x+1] <- signif(s$p.value,4)
  dose.rho[3,x+2]<- symnum(dose.rho[3,x+1], corr = FALSE, cutpoints = c(0, 0.001, 0.01, 0.05, 0.1, 1), s
  s <- with(imid, cor.test(pollen_dose[d],(hym[d]-act5c[d]), method = "spearman"))
  dose.rho[4,x] <- round(s$estimate,3)
  dose.rho[4,x+1] <- signif(s$p.value,4)
  dose.rho[4,x+2]<- symnum(dose.rho[4,x+1], corr = FALSE, cutpoints = c(0, 0.001, 0.01, 0.05, 0.1, 1), s
}
for (i in 1:length(days.factor)) {
  if (as.numeric(days.factor[i]) < 10) { days.factor[i] <- paste("0",days.factor[i],sep="") }
  }
days.factor <- paste("day",days.factor,sep="")
x <- sort(c(paste(days.factor,"rho"),paste(days.factor,"p",sep="_")))
colnames(dose.rho) <- c(x[1:2],"",x[3:4],"",x[5:6],"",x[7:8],"")
dose.rho
```

```
##     day00 rho day00_p     day09 rho  day09_p     day19 rho day19_p
## aba     0.134  0.4431         0.483 0.002839 **      0.400  0.0191 *
## apd     0.259  0.1323         0.256 0.131900         0.023  0.8991
## def    -0.102  0.5611        -0.012 0.944800         0.252  0.1497
## hym    -0.273  0.1121         0.237 0.164400         0.255  0.1450
##     day31 rho   day31_p
## aba     0.759 4.288e-05 ***
## apd     0.821 2.855e-06 ***
## def     0.405 6.136e-02 .
## hym     0.616 2.285e-03 **
```

The output above appears in the main text as Table 3.

Significant dose-dependent positive correlations were found for the expression of *abaecin* at 9 days exposure and thereafter. Correlations for *apidaecin* and *hymenoptaecin* to imidacloprid dose were also significant by day 31. At day 31, *defensin* expression was suggestive of a dose-dependent response, but the effect was not significant.

Next, we examined whether AMPs are significantly correlated in their expression among one another, across the entire dataset.

```
amp.rho <- data.frame(row.names = amp.names[2:4])
s <- with(imid, cor.test(aba-act5c,apd-act5c, method = "spearman"))
amp.rho[1,1] <- round(s$estimate,3)
amp.rho[1,2] <- signif(p.adjust(s$p.value, method="bonferroni", n=6),4)
amp.rho[1,3] <- symnum(amp.rho[1,2], corr = FALSE, cutpoints = c(0, 0.001, 0.01, 0.05, 0.1, 1), symbols
s <- with(imid, cor.test(aba-act5c,def-act5c, method = "spearman"))
amp.rho[2,1] <- round(s$estimate,3)
amp.rho[2,2] <- signif(p.adjust(s$p.value, method="bonferroni", n=6),4)
amp.rho[2,3] <- symnum(amp.rho[2,2], corr = FALSE, cutpoints = c(0, 0.001, 0.01, 0.05, 0.1, 1), symbols
s <- with(imid, cor.test(aba-act5c,hym-act5c, method = "spearman"))
amp.rho[3,1] <- round(s$estimate,3)
amp.rho[3,2] <- signif(p.adjust(s$p.value, method="bonferroni", n=6),4)
amp.rho[3,3] <- symnum(amp.rho[3,2], corr = FALSE, cutpoints = c(0, 0.001, 0.01, 0.05, 0.1, 1), symbols
s <- with(imid, cor.test(apd-act5c,def-act5c, method = "spearman"))
amp.rho[2,4] <- round(s$estimate,3)
amp.rho[2,5] <- signif(p.adjust(s$p.value, method="bonferroni", n=6),4)
amp.rho[2,6] <- symnum(amp.rho[2,5], corr = FALSE, cutpoints = c(0, 0.001, 0.01, 0.05, 0.1, 1), symbols
s <- with(imid, cor.test(apd-act5c,hym-act5c, method = "spearman"))
amp.rho[3,4] <- round(s$estimate,3)
amp.rho[3,5] <- signif(p.adjust(s$p.value, method="bonferroni", n=6),4)
amp.rho[3,6] <- symnum(amp.rho[3,5], corr = FALSE, cutpoints = c(0, 0.001, 0.01, 0.05, 0.1, 1), symbols
s <- with(imid, cor.test(def-act5c,hym-act5c, method = "spearman"))
amp.rho[3,7] <- round(s$estimate,3)
amp.rho[3,8] <- signif(p.adjust(s$p.value, method="bonferroni", n=6),4)
amp.rho[3,9] <- symnum(amp.rho[3,8], corr = FALSE, cutpoints = c(0, 0.001, 0.01, 0.05, 0.1, 1), symbols
x <- sort(c(paste(amp.names[1:3],"rho"),paste(amp.names[1:3],"p",sep="_")))
colnames(amp.rho) <- c(x[1:2],"",x[3:4],"",x[5:6],"")
amp.rho
```

```
##     aba rho     aba_p     apd rho     apd_p      def rho     def_p
## apd   0.525 1.699e-09 ***      NA        NA <NA>      NA        NA <NA>
## def   0.651 6.610e-16 ***   0.123 1.0000000           NA        NA <NA>
## hym   0.759 0.000e+00 ***   0.330 0.0009967   ***   0.732 7.759e-22  ***
```

The normalized expression of AMPs was strongly correlated among one another, except for *apidaecin* and *defensin*.

Change in AMP expression by dose

To compare the effects of different imidacloprid treatments on AMP expression, we examined the AMP expression of samples from the last (day 31) collection in comparison to the average AMP expression for bees from the same colony at the start of the experiment.

```
t0.hive.means <- data.frame(row.names = levels(imid$hivename))
t0.hive.means[,1:4] <- NA
colnames(t0.hive.means) <- colnames(imid)[7:10]
t0.hive.means[,'aba'] <- with(imid[which(imid$day==0),],by(aba-act5c,hivename,mean))
t0.hive.means[,'apd'] <- with(imid[which(imid$day==0),],by(apd-act5c,hivename,mean))
t0.hive.means[,'def'] <- with(imid[which(imid$day==0),],by(def-act5c,hivename,mean))
t0.hive.means[,'hym'] <- with(imid[which(imid$day==0),],by(hym-act5c,hivename,mean))
tn.ind <- imid[which(imid$day==31),c(2:3,5,7:10)]
tn.ind[4:7] <- tn.ind[4:7] - imid[which(imid$day==31),'act5c']
imid.c <- tn.ind; imid.c[,4:7] <- NA
for (i in 1:dim(tn.ind)[1]) {
  imid.c[i,4:7] <- tn.ind[i,4:7] - t0.hive.means[tn.ind$hivename[i],]
}
```

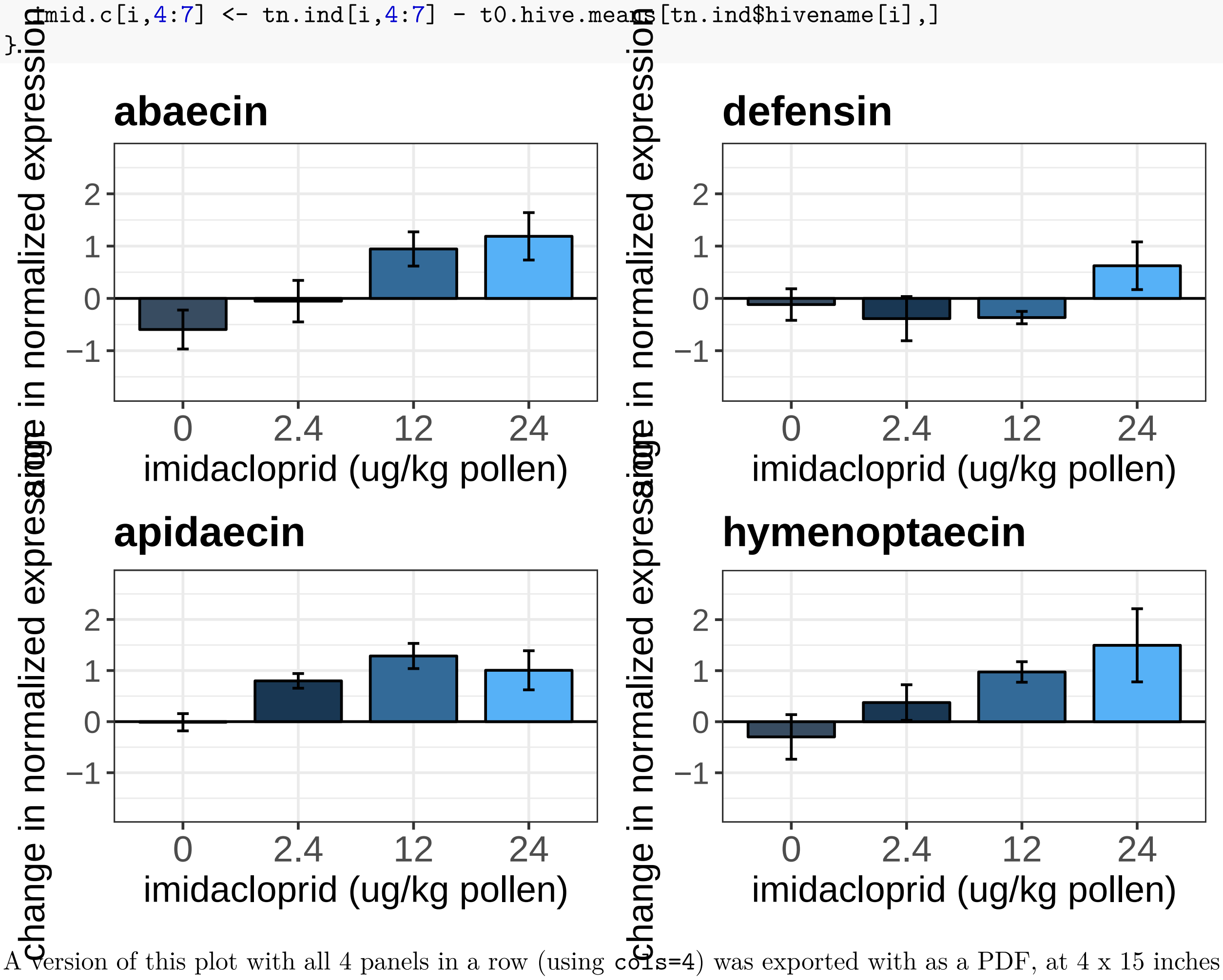

A version of this plot with all 4 panels in a row (using cols=4) was exported with as a PDF, at 4 x 15 inches (landscape), and appears in the manuscript as Figure 2E-H.

```
with(imid.c, by(aba,pollen_dose,shapiro.test))
```

```
## pollen_dose: 0
##
##  Shapiro-Wilk normality test
```

```
##
## data:  dd[x, ]
## W = 0.84319, p-value = 0.1385
##
## --------------------------------------------------------
## pollen_dose: 2.4
##
##  Shapiro-Wilk normality test
##
## data:  dd[x, ]
## W = 0.9803, p-value = 0.953
##
## --------------------------------------------------------
## pollen_dose: 12
##
##  Shapiro-Wilk normality test
##
## data:  dd[x, ]
## W = 0.83516, p-value = 0.152
##
## --------------------------------------------------------
## pollen_dose: 24
##
##  Shapiro-Wilk normality test
##
## data:  dd[x, ]
## W = 0.87458, p-value = 0.2855
```

```
with(imid.c, by(apd,pollen_dose,shapiro.test))
```

```
## pollen_dose: 0
##
##  Shapiro-Wilk normality test
##
## data:  dd[x, ]
## W = 0.71142, p-value = 0.008137
##
## --------------------------------------------------------
## pollen_dose: 2.4
##
##  Shapiro-Wilk normality test
##
## data:  dd[x, ]
## W = 0.89203, p-value = 0.3289
##
## --------------------------------------------------------
## pollen_dose: 12
##
##  Shapiro-Wilk normality test
##
## data:  dd[x, ]
## W = 0.87711, p-value = 0.2964
##
## --------------------------------------------------------
## pollen_dose: 24
```

```
##
##  Shapiro-Wilk normality test
##
## data:  dd[x, ]
## W = 0.95139, p-value = 0.7471
```

```r
with(imid.c, by(def,pollen_dose,shapiro.test))
```

```
## pollen_dose: 0
##
##  Shapiro-Wilk normality test
##
## data:  dd[x, ]
## W = 0.85118, p-value = 0.1609
##
## --------------------------------------------------------
## pollen_dose: 2.4
##
##  Shapiro-Wilk normality test
##
## data:  dd[x, ]
## W = 0.9673, p-value = 0.8738
##
## --------------------------------------------------------
## pollen_dose: 12
##
##  Shapiro-Wilk normality test
##
## data:  dd[x, ]
## W = 0.87391, p-value = 0.2826
##
## --------------------------------------------------------
## pollen_dose: 24
##
##  Shapiro-Wilk normality test
##
## data:  dd[x, ]
## W = 0.87082, p-value = 0.2698
```

```r
with(imid.c, by(hym,pollen_dose,shapiro.test))
```

```
## pollen_dose: 0
##
##  Shapiro-Wilk normality test
##
## data:  dd[x, ]
## W = 0.79587, p-value = 0.05394
##
## --------------------------------------------------------
## pollen_dose: 2.4
##
##  Shapiro-Wilk normality test
##
## data:  dd[x, ]
## W = 0.9258, p-value = 0.5481
```

```
##
## --------------------------------------------------------
## pollen_dose: 12
##
##  Shapiro-Wilk normality test
##
## data:  dd[x, ]
## W = 0.80543, p-value = 0.08966
##
## --------------------------------------------------------
## pollen_dose: 24
##
##  Shapiro-Wilk normality test
##
## data:  dd[x, ]
## W = 0.86971, p-value = 0.2653
```

```
with(imid.c, leveneTest(aba,pollen_dose))
```

```
## Levene's Test for Homogeneity of Variance (center = median)
##       Df F value Pr(>F)
## group  3  0.0926 0.9631
##       18
```

```
with(imid.c, leveneTest(apd,pollen_dose))
```

```
## Levene's Test for Homogeneity of Variance (center = median)
##       Df F value Pr(>F)
## group  3  0.9463 0.4391
##       18
```

```
with(imid.c, leveneTest(def,pollen_dose))
```

```
## Levene's Test for Homogeneity of Variance (center = median)
##       Df F value Pr(>F)
## group  3  1.2778  0.312
##       18
```

```
with(imid.c, leveneTest(hym,pollen_dose))
```

```
## Levene's Test for Homogeneity of Variance (center = median)
##       Df F value Pr(>F)
## group  3  0.4179 0.7423
##       18
```

Only *apidaecin* values for the control treatment are significantly different from normality, but given the low sample sizes, we will use non-parametric tests to compare the changes among treatments.

```
kruskal.test(aba~pollen_dose, data=imid.c)
```

```
##
##  Kruskal-Wallis rank sum test
##
## data:  aba by pollen_dose
## Kruskal-Wallis chi-squared = 11.191, df = 3, p-value = 0.01074
```

```
kruskal.test(apd~pollen_dose, data=imid.c)
```

```
##
```

```
##  Kruskal-Wallis rank sum test
##
## data:  apd by pollen_dose
## Kruskal-Wallis chi-squared = 12.281, df = 3, p-value = 0.006479
```

```r
kruskal.test(def~pollen_dose, data=imid.c)
```

```
##
##  Kruskal-Wallis rank sum test
##
## data:  def by pollen_dose
## Kruskal-Wallis chi-squared = 3.8704, df = 3, p-value = 0.2758
```

```r
kruskal.test(hym~pollen_dose, data=imid.c)
```

```
##
##  Kruskal-Wallis rank sum test
##
## data:  hym by pollen_dose
## Kruskal-Wallis chi-squared = 8.2996, df = 3, p-value = 0.04021
```

Significant differences exist for *abaecin*, *apidaecin*, and *hymenoptaecin*.

```r
with(imid.c, dunn.test(aba, g=pollen_dose, method="bonferroni"))
```

```
##   Kruskal-Wallis rank sum test
##
## data: aba and pollen_dose
## Kruskal-Wallis chi-squared = 11.1905, df = 3, p-value = 0.01
##
##
##                          Comparison of aba by pollen_dose
##                                    (Bonferroni)
## Col Mean-|
## Row Mean |          0        2.4         12
## ---------+---------------------------------
##      2.4 |  -0.844653
##          |     1.0000
##          |
##       12 |  -2.585581  -1.780236
##          |     0.0292     0.2251
##          |
##       24 |  -2.789037  -1.983692  -0.194793
##          |     0.0159     0.1419     1.0000
```

```r
with(imid.c, dunn.test(apd, g=pollen_dose, method="bonferroni"))
```

```
##   Kruskal-Wallis rank sum test
##
## data: apd and pollen_dose
## Kruskal-Wallis chi-squared = 12.2814, df = 3, p-value = 0.01
##
##
##                          Comparison of apd by pollen_dose
##                                    (Bonferroni)
## Col Mean-|
## Row Mean |          0        2.4         12
```

```
## ----------+-----------------------------------
##      2.4 |  -2.089404
##          |     0.1100
##          |
##       12 |  -3.314630  -1.322461
##          |     0.0028     0.5580
##          |
##       24 |  -2.500808  -0.508638   0.779175
##          |     0.0372     1.0000     1.0000
```

```
with(imid.c, dunn.test(hym, g=pollen_dose, method="bonferroni"))
```

```
##   Kruskal-Wallis rank sum test
##
## data: hym and pollen_dose
## Kruskal-Wallis chi-squared = 8.2996, df = 3, p-value = 0.04
##
##
##                        Comparison of hym by pollen_dose
##                                  (Bonferroni)
## Col Mean-|
## Row Mean |          0        2.4         12
## ----------+-----------------------------------
##      2.4 |  -1.155840
##          |     0.7432
##          |
##       12 |  -1.890441  -0.788390
##          |     0.1761     1.0000
##          |
##       24 |  -2.755127  -1.653076  -0.827874
##          |     0.0176     0.2949     1.0000
```

Colony mass

To compare the mass of colonies in each treatment at the completion of the experiment, we used the Kruskal-Wallis rank sum test due to the sample size in each treatment.

```
hive.mass <- read.csv("Bi.hive.masses.csv", header = T)
kruskal.test(grams_nestcage~treatment, data=hive.mass)
```

```
##
##  Kruskal-Wallis rank sum test
##
## data:  grams_nestcage by treatment
## Kruskal-Wallis chi-squared = 4.5673, df = 3, p-value = 0.2064
```

No significant difference among treatments is apparant.

Mock experiment

```
mock <- read.csv("Bi.mock.treatment.timecourse.csv", header = T)
```

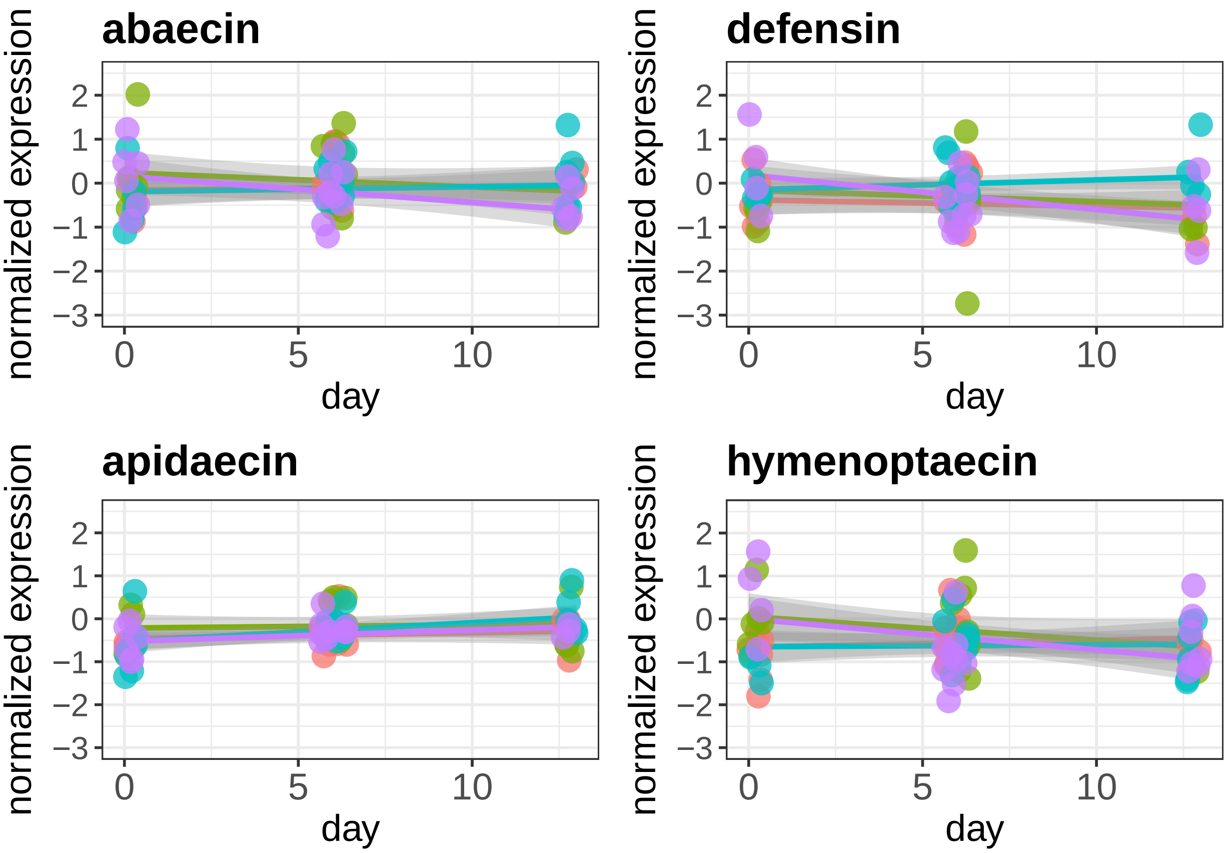

A version of this plot with all 4 panels in a row (using `cols=4`) was exported with as a PDF, at 4 x 15 inches (landscape), and appears in the manuscript as Supplementary Figure S1.

We tested whether change in AMP expression over the course of the mock experiment differed among the pseudo-treatment blocks.

```
mock.t0.hive.means <- data.frame(row.names = levels(mock$hive))
mock.t0.hive.means[,1:4] <- NA
colnames(mock.t0.hive.means) <- colnames(mock)[5:8]
mock.t0.hive.means[,'aba'] <- with(mock[which(mock$day==0),],by(aba-act5c,hive,mean))
mock.t0.hive.means[,'apd'] <- with(mock[which(mock$day==0),],by(apd-act5c,hive,mean))
mock.t0.hive.means[,'def'] <- with(mock[which(mock$day==0),],by(def-act5c,hive,mean))
mock.t0.hive.means[,'hym'] <- with(mock[which(mock$day==0),],by(hym-act5c,hive,mean))
mock.tn.ind <- mock[which(mock$day==13),c(2:3,5:8)]
mock.tn.ind[3:6] <- mock.tn.ind[3:6] - mock[which(mock$day==13),'act5c']
mock.c <- mock.tn.ind; mock.c[,3:6] <- NA
for (i in 1:dim(tn.ind)[1]) {
  mock.c[i,3:6] <- mock.tn.ind[i,3:6] - mock.t0.hive.means[mock.tn.ind$hive[i],]
}
kruskal.test(aba~block, data=mock.c)
```

```
##
##  Kruskal-Wallis rank sum test
##
## data:  aba by block
## Kruskal-Wallis chi-squared = 6.1976, df = 3, p-value = 0.1024
```

```r
kruskal.test(apd~block, data=mock.c)
```

```
##
##  Kruskal-Wallis rank sum test
##
## data:  apd by block
## Kruskal-Wallis chi-squared = 5.1739, df = 3, p-value = 0.1595
```

```r
kruskal.test(def~block, data=mock.c)
```

```
##
##  Kruskal-Wallis rank sum test
##
## data:  def by block
## Kruskal-Wallis chi-squared = 9.9038, df = 3, p-value = 0.0194
```

```r
kruskal.test(hym~block, data=mock.c)
```

```
##
##  Kruskal-Wallis rank sum test
##
## data:  hym by block
## Kruskal-Wallis chi-squared = 6.2569, df = 3, p-value = 0.09976
```

```r
with(mock.c, dunn.test(def, block, method = "bonferroni"))
```

```
##   Kruskal-Wallis rank sum test
##
## data: def and block
## Kruskal-Wallis chi-squared = 9.9038, df = 3, p-value = 0.02
##
##
##                         Comparison of def by block
##                                (Bonferroni)
## Col Mean-|
## Row Mean |          A          B          C
## ---------+---------------------------------
##        B |   0.711286
##          |     1.0000
##          |
##        C |  -1.769377  -2.548553
##          |     0.2305     0.0325
##          |
##        D |   1.306933   0.926734   2.223897
##          |     0.5737     1.0000     0.0785
```

The expression changes over time of *abaecin*, *apidaecin* and *hymenoptaecin* did not differ significantly among the four blocks. There was a significant difference between two blocks in the expression change over time for *defensin*, which did not differ in the imidacloprid experiment.

```r
with(mock.c, boxplot(def~block, xlab="block", ylab="change in normalized expression"))
```

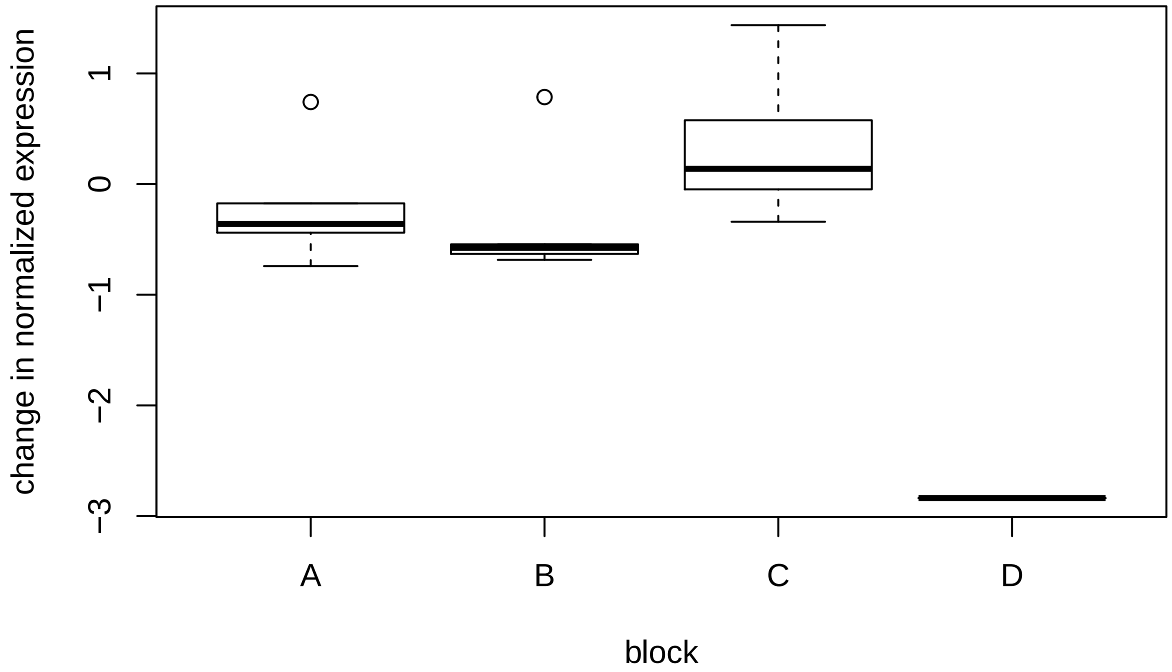

Wild *B. impatiens*

```
wild <- read.csv("Bi.wild.csv", header = T)
captives <- rbind(imid[which(imid$day==0 | imid$pollen_dose==0),7:11], mock[,5:9])
captives <- cbind(rep("screenhouse",dim(captives)[1]),captives)
colnames(captives)[1] <- c("locality")
wild.comp <- rbind(wild[,2:7], captives)
```

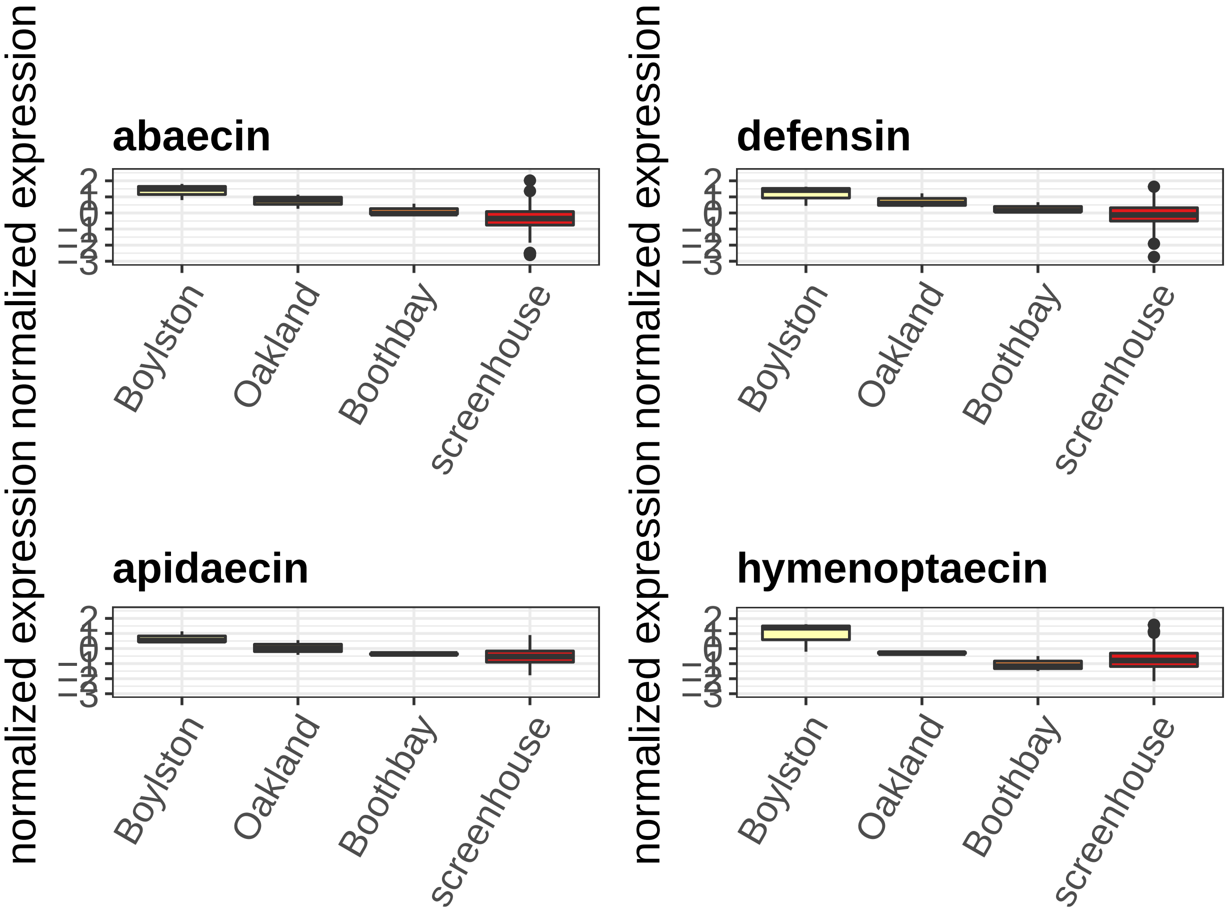

A version of this plot with all 4 panels in a row (using `cols=4`) was exported with as a PDF, at 5 x 15 inches (landscape), and appears in the manuscript as Figure 3.

Samples sizes differ strongly. Therefore, we tested for differences among wild and captives bees using nonparametric tests.

```
kruskal.test(aba-act5c~locality,data=wild.comp)
```

```
##
##  Kruskal-Wallis rank sum test
##
## data:  aba - act5c by locality
## Kruskal-Wallis chi-squared = 15.213, df = 3, p-value = 0.001643
```

```
kruskal.test(apd-act5c~locality,data=wild.comp)
```

```
##
##  Kruskal-Wallis rank sum test
##
## data:  apd - act5c by locality
## Kruskal-Wallis chi-squared = 11.287, df = 3, p-value = 0.01027
```

```
kruskal.test(def-act5c~locality,data=wild.comp)
```

```
##
##  Kruskal-Wallis rank sum test
##
## data:  def - act5c by locality
## Kruskal-Wallis chi-squared = 11.925, df = 3, p-value = 0.007643
```

```
kruskal.test(hym-act5c~locality,data=wild.comp)
```

```
##
##  Kruskal-Wallis rank sum test
##
## data:  hym - act5c by locality
## Kruskal-Wallis chi-squared = 9.5206, df = 3, p-value = 0.02311
```

```
with(wild.comp,dunn.test(aba-act5c, locality, method = "bonferroni"))
```

```
##   Kruskal-Wallis rank sum test
##
## data: x and locality
## Kruskal-Wallis chi-squared = 15.2132, df = 3, p-value = 0
##
##
##                           Comparison of x by locality
##                                  (Bonferroni)
## Col Mean-|
## Row Mean |     Boylst    Oakland   Boothbay
## ---------+---------------------------------
##  Oakland |   0.315192
##          |     1.0000
##          |
## Boothbay |   1.053407   0.738214
##          |     0.8765     1.0000
##          |
## screenho |   2.842091   2.400437   1.366037
##          |     0.0134     0.0491     0.5158
```

```
with(wild.comp,dunn.test(apd-act5c, locality, method = "bonferroni"))
```

```
##   Kruskal-Wallis rank sum test
##
## data: x and locality
## Kruskal-Wallis chi-squared = 11.2865, df = 3, p-value = 0.01
##
##
##                           Comparison of x by locality
##                                  (Bonferroni)
## Col Mean-|
## Row Mean |     Boylst    Oakland   Boothbay
## ---------+---------------------------------
##  Oakland |   0.713330
##          |     1.0000
##          |
## Boothbay |   1.609141   0.895810
##          |     0.3228     1.0000
##          |
## screenho |   2.817186   1.817653   0.562426
##          |     0.0145     0.2074     1.0000
```

```
with(wild.comp,dunn.test(def-act5c, locality, method = "bonferroni"))
```

```
##   Kruskal-Wallis rank sum test
##
```

```
## data: x and locality
## Kruskal-Wallis chi-squared = 11.9254, df = 3, p-value = 0.01
##
##
##                            Comparison of x by locality
##                                    (Bonferroni)
## Col Mean-|
## Row Mean |     Boylst    Oakland   Boothbay
## ---------+---------------------------------
##  Oakland |   0.273720
##          |     1.0000
##          |
## Boothbay |   0.937284   0.663564
##          |     1.0000     1.0000
##          |
## screenho |   2.514860   2.131318   1.201519
##          |     0.0357     0.0992     0.6886
```

```
with(wild.comp,dunn.test(hym-act5c, locality, method = "bonferroni"))
```

```
##   Kruskal-Wallis rank sum test
##
## data: x and locality
## Kruskal-Wallis chi-squared = 9.5206, df = 3, p-value = 0.02
##
##
##                            Comparison of x by locality
##                                    (Bonferroni)
## Col Mean-|
## Row Mean |     Boylst    Oakland   Boothbay
## ---------+---------------------------------
##  Oakland |   0.713330
##          |     1.0000
##          |
## Boothbay |   2.446890   1.733559
##          |     0.0432     0.2490
##          |
## screenho |   2.525324   1.525792  -0.903304
##          |     0.0347     0.3812     1.0000
```

While, examinng lanscape-level variation in AMP expression was not a focus of this study, we were curious if differences among localities correlated with any obvious factors, such as local human population density (based on 2010 US Census data for each municipality), latitude or longitude.

```
with(wild,cor.test(aba-act5c,popdens,method = "spearman"))$p.value
```

```
## [1] 0.1970221
```

```
with(wild,cor.test(apd-act5c,popdens,method = "spearman"))$p.value
```

```
## [1] 0.3285304
```

```
with(wild,cor.test(def-act5c,popdens,method = "spearman"))$p.value
```

```
## [1] 0.4070838
```

```
with(wild,cor.test(hym-act5c,popdens,method = "spearman"))$p.value
```

```
## [1] 0.1448393
```

```r
with(wild,cor.test(aba-act5c,lat,method = "spearman"))$p.value
```

```
## [1] 0.4070838
```

```r
with(wild,cor.test(apd-act5c,lat,method = "spearman"))$p.value
```

```
## [1] 0.3285304
```

```r
with(wild,cor.test(def-act5c,lat,method = "spearman"))$p.value
```

```
## [1] 0.4070838
```

```r
with(wild,cor.test(hym-act5c,lat,method = "spearman"))$p.value
```

```
## [1] 0.3285304
```

No significant correlations to normalized AMP expression were found with population density or latitude.

```r
with(wild,cor.test(aba-act5c,lon,method = "spearman"))
```

```
##
##  Spearman's rank correlation rho
##
## data:  aba - act5c and lon
## S = 214.87, p-value = 0.0112
## alternative hypothesis: true rho is not equal to 0
## sample estimates:
##        rho
## -0.7905694
```

```r
with(wild,cor.test(apd-act5c,lon,method = "spearman"))
```

```
##
##  Spearman's rank correlation rho
##
## data:  apd - act5c and lon
## S = 208.54, p-value = 0.02324
## alternative hypothesis: true rho is not equal to 0
## sample estimates:
##        rho
## -0.7378648
```

```r
with(wild,cor.test(def-act5c,lon,method = "spearman"))
```

```
##
##  Spearman's rank correlation rho
##
## data:  def - act5c and lon
## S = 195.89, p-value = 0.06758
## alternative hypothesis: true rho is not equal to 0
## sample estimates:
##        rho
## -0.6324555
```

```r
with(wild,cor.test(hym-act5c,lon,method = "spearman"))
```

```
##
##  Spearman's rank correlation rho
```

```
##
## data:  hym - act5c and lon
## S = 227.52, p-value = 0.001078
## alternative hypothesis: true rho is not equal to 0
## sample estimates:
##        rho
## -0.8959787
```

Expression of all 4 AMPs in wild *B. impatiens* was significantly correlated with longitude. However, this may be driven largely by the fact that while Oakland and Boothbay, Maine are relatively close in their longitude, Boylston, Massachusetts is dramatically farther west.

```
wild[c(4,7,1),c(2,8:10)]
```

```
##     locality      lat       lon popdens
## 4   Boylston 42.36258 -71.72631   220.0
## 7    Oakland 44.57276 -69.68694   243.1
## 1   Boothbay 43.87400 -69.66252   142.3
```
